# Evaluation of risk-based travel policy for the COVID-19 epidemic in Scotland: a population-based surveillance study

**DOI:** 10.1136/bmjopen-2024-085332

**Published:** 2024-11-29

**Authors:** Isobel McLachlan, Selene Huntley, Kirstin Leslie, Jennifer Bishop, Christopher Redman, Gonzalo Yebra, Sharif Shaaban, Nicolaos Christofidis, Samantha Lycett, Matthew T G Holden, David L Robertson, Alison Smith-Palmer, Joseph Hughes, Sema Nickbakhsh

**Affiliations:** 1 Public Health Scotland, Edinburgh, UK; 2 The Roslin Institute, University of Edinburgh, Edinburgh, UK; 3 School of Medicine, University of St Andrews, St Andrews, UK; 4 MRC-University of Glasgow Centre for Virus Research, Glasgow, UK; 5 School of Biodiversity, One Health & Veterinary Medicine, University of Glasgow, Glasgow, UK

**Keywords:** Health policy, SARS-CoV-2 Infection, Risk Factors, Health informatics, Public health, Epidemiology

## Abstract

**Abstract:**

**Objectives:**

We aimed to assess the effects of risk-based travel restrictions on (1) international travel frequency, (2) SARS-CoV-2 case importation risk, (3) national SARS-CoV-2 incidence and (4) importation of SARS-CoV-2 variants into Scotland.

**Design:**

Population-based surveillance study.

**Setting:**

The study utilises SARS-CoV-2 community testing from February 2021 to May 2022 in Scotland, UK and spans the introduction of the UK’s ‘traffic light system’ policy in May 2021.

**Primary outcome measures:**

Travel-related cases of COVID-19 were defined as PCR-positive Scottish residents self-reporting international travel within 14 days of booking a postarrival travel test. The Red-Amber-Green (RAG) status of the reported travel destination was determined through data linkage using country and date.

**Results:**

International flight passengers arriving into Scotland increased by 754% during the traffic light period. Amber list countries were the most frequently visited and ranked highly for both SARS-CoV-2 importations and contribution to national case incidence. Rates of international travel and associated SARS-CoV-2 case rates varied significantly across age, health board and deprivation groups. Multivariable logistic regression revealed SARS-CoV-2 case detections were less likely through travel-based than community-based surveillance systems, although increased from green-to-amber and amber-to-red lists. When examined according to travel destination, SARS-CoV-2 importation risks did not strictly follow RAG designations, and red lists did not prevent establishment of novel SARS-CoV-2 variants.

**Conclusions:**

Our findings suggest that country-specific postarrival screening undertaken in Scotland did not prohibit the public health impact of COVID-19 in Scotland. Travel rates likely contributed to patterns of SARS-CoV-2 case importation and population incidence.

**Strengths and limitations of this study**

- This study combines extensive national data sets on international flight arrivals into Scotland, clinical microbiological data and SARS-CoV-2 genomic data.
- Individuals with recent international travel were identifiable through national surveillance and linked to Red-Amber-Green status of the travel destination.
- SARS-CoV-2 tests were stratified by week, demographic factors including deprivation and the travel and RAG status of the travel destination.
- There were an unknown number of individuals exempt from postarrival SARS-CoV-2 PCR testing and levels of compliance are not known.
- Relative SARS-CoV-2 case detections across travel-based and community-based surveillance reflect, in part, differences in surveillance design.

## Background

 On 24 March 2020 Scotland, with the rest of the UK, entered a national lockdown to limit SARS-CoV-2 spread and subsequent COVID-19-associated hospitalisations. This early period of the pandemic was characterised by British nationals being advised against all but essential travel,^1^ alongside unprecedented border closures implemented increasingly by countries globally.^2^ However, prior to implementation of these stringent travel restrictions in the UK, hundreds of introductions of SARS-CoV-2 into Scotland had already occurred through international travel and community transmission was underway.^3^

By July 2020, circulating SARS-CoV-2 was dramatically reduced^4^ and Scotland had moved to phase 3 of a route map out of lockdown.^5^ While interventions focusing on reducing social contacts remained in place, travel restrictions were relaxed with the lifting of quarantine measures from certain overseas destinations.^6^ A country-specific risk assessment approach was subsequently undertaken with the introduction of ‘travel corridors’, relaxing self-isolation measures for individuals travelling to countries not considered to be high risk for high levels of SARS-CoV-2 exposure.^7^

Progress out of lockdown was short lived however, with increasing COVID-19 incidence linked to travel in the summer of 2020^4 8^ and the subsequent identification of the first novel SARS-CoV-2 Variant of Concern (VOC), Alpha (B.1.1.7 Pango lineage^9^), in Scotland during November 2020. This variant was first identified in the UK and classified a VOC based on risk assessment.^10^ Scotland subsequently entered a period with travel restrictions enforced at a local authority level based on regional infection rates, regulating both domestic and international travel, and the country entered its second national lockdown on 5 January 2021. By 4 January 2021, Alpha was the dominant variant circulating in the UK and managing the trade-off between COVID-19 hospitalisations and recovery of the economy including the travel industry was of high concern. Other variants of concern were also identified, such as Beta (B.1.351) in South Africa, resulting in a long-standing travel ban to this country.

Subsequently, in line with the lifting of lockdown restrictions and safe resumption of international travel, May 2021 marked the introduction of a ‘traffic light system’ across the UK (in place 17 May 2021 to 30 September 2021) which applied relaxed quarantine and testing requirements for travellers on a country-specific basis through Red-Amber-Green (RAG) list designations.^11^

During this period, the epidemiological course of the epidemic shifted in the UK, with the importation of a fourth novel VOC, Delta (B.1.617.2; first identified in India),^12^ although widespread rollout of the national COVID-19 vaccination programme was also in place at this time. Delta replaced Alpha as the dominant variant in Scotland by the end of May 2021.^13^ The traffic light system was subsequently lifted across the UK on 4 October 2021, with Scotland adopting a simplified system of ‘red list’ and ‘not red’.^14^ However, a fifth novel VOC, Omicron (the BA.1 sublineage; first identified in South Africa), was identified in Scotland at the end of November 2021.^15 16^ Several countries deemed high risk were given red list status in an attempt to slow the introduction and spread of Omicron in the UK,^17^ although this highly transmissible variant was soon established in the community. Travel regulations were eventually lifted incrementally and fully removed across the UK on 18 March 2022.^18^

International travel continues to present the main risk pathway for importations of novel SARS-CoV-2 variants of public health significance. In the UK, a temporary increase in surveillance was reintroduced between 5 January 2023 and 5 April 2023 for individuals travelling from specific locations, owing to the evolving epidemiological situation of other nations experiencing increased COVID-19 activity and the emergence of further variants of potential public health significance.^19^ There is, however, a need for improved understanding of how risks of novel SARS-CoV-2 introductions and subsequent spread vary by the geography and demography of populations, to inform proportionate travel-related interventions and early warning surveillance systems. There have been few observational studies providing evidence on the public health effects of COVID-19 travel restrictions, with the majority focused on border closures put in place to curb global spread early in the pandemic.^2 20 21^

Here we use Scottish community surveillance and SARS-CoV-2 whole genome sequence data to evaluate the public health effects of the COVID-19 traffic light travel policy in Scotland. Our objectives were to quantify the effect of the traffic light policy on (1) international travel frequency, (2) temporal, demographic and geographic patterns of SARS-CoV-2 importation risk, (3) national SARS-CoV-2 case incidence and (4) the risk of importing specific SARS-CoV-2 VOC across RAG groups. This evaluation has importance for providing an evidence-based approach to future public health guidance and decision making around international travel policy.

## Methods

### Data sets

#### Civil Aviation Authority data

Data on the monthly number of passenger arrivals into Scottish airports, by country and airport of origin, were provided by the Civil Aviation Authority (CAA).^22^ There were 5508 records capturing 9 383 947 passengers in total from the period April 2019 to September 2021, from flights arriving into 11 Scottish airports from 290 airports in 70 countries. Data were grouped, providing total monthly numbers of flights into Scottish airports by country of origin.

#### COVID-19 Passenger Locator Forms

From June 2020, measures were introduced requiring all UK arrivals to complete a Passenger Locator Form (PLF) to support compliance with COVID-19 travel measures.^23^ The data set contained weekly data for the period 28 June 2020 to 19 March 2022 on the number of PLFs submitted to Border Control and the originating countries from which passengers had travelled into Scotland. Over the 90 week period, passengers arrived into Scotland from 247 countries.

Data on the Canary Islands and Madeira were merged with that of Spain and Portugal, respectively, in both the CAA and the PLFs to enable like-for-like country-level comparison with PCR data which did not stratify at this level. The RAG status of mainland Spain and Portugal was used as this accounted for the majority of returning travellers from these countries.

#### Surveillance of COVID-19 in Scotland

All reported SARS-CoV-2 test results in Scotland were accessed from the NHS Scotland Corporate Data Warehouse (CDW), a database designed to support the analysis and reporting of COVID-19 surveillance in Scotland. In line with the national case definition, respiratory specimens testing positive by reverse transcription real-time PCR tests 90 days or more after an individual’s last positive specimen were captured as a separate episode of infection (reinfection). The data set captured demographics (age, sex and postcode location) and test information (date of sample and test result). Reasons for testing included diagnostic confirmation for those with symptoms, asymptomatic testing of close contacts to support self-isolation, workplace testing as part of the NHS Test and Protect system and postarrival testing of travellers returning from international destinations.

#### Postarrival international travel tests

The CDW database captured postarrival international travel-related PCR tests from 15 February 2021. During the traffic light period, tests were taken on or before day 2 (RAG list countries) and on or after day 8 (red and amber list countries). When booking a postarrival COVID-19 test, individuals who had travelled internationally within the preceding 14 days were required to self-report the main country they had travelled to. All individuals were required to take postarrival tests until this requirement was incrementally removed from 7 January 2022.

There were 425 764 SARS-CoV-2 PCR test records from Scottish residents reporting recent international travel over the study period (15 February 2021 to 3 May 2022). Deduplication of SARS-CoV-2 tests was conducted by grouping multiple tests based on a unique identifier (Community Health Index) for individuals with specimen dates falling within a 14-day period and relating to the same travel destination, thereby defining ‘travel events’ by country. Following this process, 345 045 travel records remained representing 290 044 individuals. Over the study period, 41 934 (14.5%) of individuals had more than one travel event, with a minority of individuals (n=1178, 0.4%) reporting travel to more than one country within a 14-day period. Tests with specimen dates out with a 90-day period were deemed a separate episode of infection (following the national case definition^24^). Positive test records were preferentially retained over negative records for each travel event. Monthly PCR test frequencies for travellers were strongly correlated with numbers of passengers into Scotland based on PLFs (online supplemental file S1).

#### SARS-CoV-2 whole genome sequencing data

Whole Genome Sequencing (WGS) of SARS-CoV-2 samples was introduced in Scotland in March 2020. Following emergence of the Alpha variant, a push to sequence all positive SARS-CoV-2 samples was stepped up to allow for surveillance of variants, though with prioritisation towards travel-related samples to identify importation of VOCs. WGS results were uploaded by sequencing laboratories and quality assessed via the COVID-19 Genomics UK (COG-UK) pipeline hosted on the Cloud Infrastructure for Microbial Bioinformatics (CLIMB) as part of COG-UK.^25^ Definitions based on mutational patterns were then applied to resultant genomic information to designate a variant^9^ and these results were linked to the original PCR test via a unique specimen identifier.

During the study period, there were 317 570 samples from COVID-19 cases resident in Scotland that underwent SARS-CoV-2 WGS. Samples with a low-quality genome, or not linkable to a positive SARS-CoV-2 PCR test and therefore unconfirmed as a Scottish sample, were excluded (n=78). The distributions of age, sex, NHS board and Scottish Index of Multiple Deprivation (SIMD) among cases that underwent WGS were similar to total cases during the study period (online supplemental file S2).

#### Traffic light system country designations

The RAG status of each travel destination was extracted from Scottish Government online travel updates.^18^ Test records, including genome sequences, were linked to RAG status based on the recorded travel destination and the specimen date of the test. A small proportion (0.2%) of records related to travel to countries were not assigned a RAG status and were therefore excluded. Analyses aggregating data on a weekly basis applied the country’s RAG status pertaining to the start of each week, all other analyses applied the daily designations.

### Statistical analysis

#### Assessment of travel patterns by time, location and demography

Descriptive statistical analyses were performed to characterise key trends of international travel for the Scottish population before and during the pandemic, based on CAA and PLF data. Changes in the count of passengers entering Scotland, at or between key time points, were used as an objective indicator of the impact of travel-related measures on travel.

To assess the impact of the traffic light system on the risk of SARS-CoV-2 importations, trends in the weekly numbers and proportions of PCR-positive travellers were examined for the top 30 most frequently visited travel destinations, spanning a period before (February 2021–May 2021), during (May 2021–October 2021) and after (October 2021–May 2022) the traffic light system. Although postarrival tests were a mandatory travel policy, some exceptions were in place and compliance was not quantified.^23^ PLF data were therefore used to assess how representative the PCR test data was of travel frequency by destination during the traffic light period.

Groups at risk of importing SARS-CoV-2 through international travel were also assessed by comparing travel frequencies across demographic (age, sex, relative deprivation of residential location, as measured by SIMD) and geographical (territorial NHS Board) factors. The Kruskal-Wallis test was used to assess differences in mean travel frequencies across demographic and geographic groups, with a p-value<0.05 applied to indicate statistical significance. This non-parametric method was used as the Q–Q plots of the corresponding analysis of variance model residuals indicated some deviation of the data from a normal distribution. Overall results and conclusions were however comparable across these methods.

The potential impact of travel on the epidemiological situation in Scotland was assessed. To do so, numbers of travel-related SARS-CoV-2 cases were quantified as a proportion of all observed community cases of SARS-CoV-2 in Scotland. Ranking of travel destinations according to travel frequency, importation risk and impact gave an overall assessment of risk, shown for a period of low (June 2021) and high (September 2021) travel frequency.

#### Logistic regression modelling

The association between travel-based versus community-based surveillance and SARS-CoV-2 case detections was assessed in a test-negative case–control design for the traffic light system period (17 May 2021 to 30 September 2021).

There were 200 014 deduplicated travel records for this time period, and among 199 709 with information available, 2.0% had self-reported symptoms. Records for individuals without international travel over the same timeframe (n=4 011 152) were deduplicated as for the travel records. Those with specimen dates out with 90 days of the first identified records were presumed a separate episode of infection, retaining a single record per episode. Positive test results were retained over negative test results. Test records without a unique identifier were assumed to be from different individuals. Following deduplication, there were 1 891 876 non-travel records during the traffic light period, and among 1 499 891 tests with the information, 43.3% had self-reported symptoms.

The combined data set of 2 091 890 records was linked to the RAG status according to the travel destination of each record. A ‘travel status’ variable was created whereby those with an international travel event were grouped by level of RAG status and with the remaining forming a ‘non-traveller’ group.

Logistic regression modelling was undertaken, adjusting for demographic, geographic and temporal factors. Following the exclusion of a small number (5.1%) of records with incomplete information across all variables, analyses were conducted on 1 984 272 test events. Unadjusted ORs were initially quantified in univariate binary logistic regression models examining associations between SARS-CoV-2 infection and travel status, age group (0–19y, 20–39y, 40–59y, 60–79y, 80y+), sex (male vs female), month (May, June, July, August September) and NHS Board (fourteen territorial health boards).

Multivariable mixed-effects logistic regression modelling was then used to quantify the relative odds of SARS-CoV-2 case detection adjusting for age group, sex and calendar month as fixed effects and NHS Board location as a random effect. Statistical interactions were assessed for all factors that were significant in the final multivariable model, with a p-value less than 0.05 indicating statistical significance. A small number of individuals (n=8990, 0.4%) had a subsequent record (positive and/or negative tests) outwith the 90-day episode window. Restricting analyses to the first observed records per individual did not alter the magnitude, direction or significance of the estimated ORs.

All analyses were performed in R V.3.6.1.^26^ The glm function was applied for single-effect statistical models and the lme4 (1.1–27.1 package) for mixed-effect models.

### Patient and public involvement

Patients and/or the public were not involved in the design, conduct, reporting, or dissemination plans of this study.

## Results

### Impact of COVID-19 policies on patterns of international travel in Scotland

**Figure 1:**
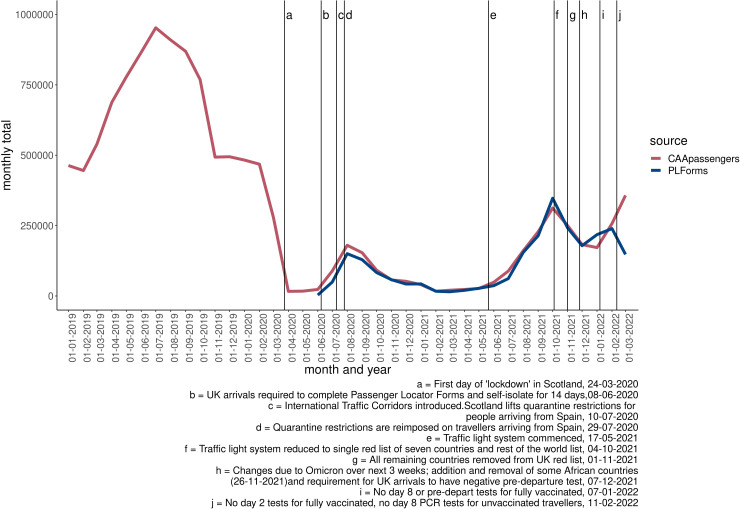
Monthly numbers of passengers into Scotland, before and during the pandemic, January. Data from Civil Aviation Authority data and Passenger Locator forms spanning January 2019 to March 2022. CAA, Civil Aviation Authority; PL, Passenger Locator.

Prior to the COVID-19 pandemic, numbers of international flight passengers arriving into Scotland per month were estimated to range from around 445 000 passengers (February 2019) to 952 000 (July 2019) during the peak summer period (CAA data, see figure 1). During the COVID-19 pandemic, monthly numbers of international flight passengers decreased substantially owing to travel restrictions (figure 1). A reduction in travel of 97.7% (to around 17 000 passengers per month, compared with the same period in 2019) was observed during the first national lockdown (April to May, 2020), by 86.7% (to around 95 000 passengers per month, compared with the same period in 2019) during a period of relaxed travel restrictions (data from July 2020 to January, 2021), and by 87.4% (to around 111 000 passengers per month, compared with the same period in 2019) during the period of the traffic light system (May to September 2021).

**Figure 2:**
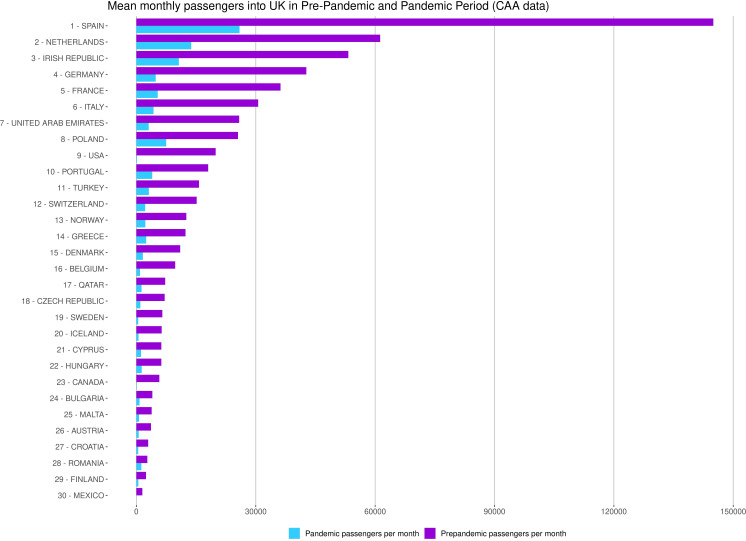
Mean monthly number of passengers with Scottish residence travelling into the UK. Based on Civil Aviation Authority data comparing periods before (January 2019 to February 2020) and during (March 2020–March 2022) the COVID-19 pandemic. CAA, Civil Aviation Authority.

The magnitude of reduced international travel across the phases of COVID-19 travel restrictions was largely consistent across destinations and data sources (online supplemental file S3). Despite large reductions in travel, the most frequently visited destinations remained largely consistent however, with Spain the most common destination both prior to and during the COVID-19 pandemic (figure 2).

The traffic light system (17 May 2021 to 4 October 2021) followed a country-specific risk-based approach (see online supplemental file S4), with quarantine reserved for high-risk countries (red list), and others requiring self-isolation together with days 2 and 8 postarrival tests (amber list), or day-2 postarrival tests only (green list). The frequency of international arrivals into Scottish airports subsequently increased by 754% from May 2021 to September 2021, compared with a 12% increase over the same period in 2019 (figure 1).

The ranking of travel frequency across RAG groups remained consistent over time when retrospectively applying designations to the prepandemic period, with amber list countries the most frequently visited (83.8% and 70.0% in the pandemic period based on PLF and CAA data sources, respectively, and 74.8% prepandemic (CAA data)). A small discrepancy was seen between red and green list countries although they were largely consistent throughout the traffic light period (online supplemental file S4). The largest decrease in travel frequency, comparing May-to-September inclusive prepandemic and-postpandemic, was seen for red list (87.7% decrease) followed by amber (78.8% decrease) and green list (63.2% decrease) countries (online supplemental file S4).

Despite the lifting of the traffic light system and incremental removal of travel restrictions in winter 2021/2022, the frequency of international travel declined during this period, consistent with the typical seasonality of travel (figure 1).

### Travel and case patterns by demographic and geographic groups

There were 1 356 983 SARS-CoV-2 positive case records in total for the study period of 15 February 2021 to 3 May 2022. This period spanned the introduction of postarrival tests for travellers returning from international destinations to a period postlifting of travel restrictions. Within this time period, there were 25 154 SARS-CoV-2 positive cases among 345 045 international travel events.

When assessing variation in travel-related SARS-CoV-2 detections, a significant difference in average weekly rates of travel was found by age group, deprivation and NHS Board, but not for sex. A similar pattern was observed for SARS-CoV-2 detection rates among travellers (table 1). Both travel and SARS-CoV-2 case detection rates were notably higher in working-age adults (age groups 20–39y and 40–59y), for residents of NHS Boards with larger populations, and increased with decreasing deprivation; over half of all international travel events were from individuals residing in the lowest deprivation groups.

**Table 1:** Frequency of international travel events across demographic and geographical groups, during the period of the traffic light system (17 May 2021 to 26 September 2021) in Scotland

Factor	Level	Mean weekly travel events per 10 000 population	Kruskal-Wallis test, p value*	Mean weekly cases among travellers per 10 000 population	Kruskal-Wallis test, p value*
Age group	0–19y	5.98	p<0.001	0.25	p<0.001
	20–39y	26.31		0.40	
	40–59y	21.69		0.29	
	60–79y	12.36		0.12	
	80y+	1.73		0.05	
Sex	Male	17.29	p=0.569	0.28	p=0.343
	Female	16.13		0.24	
NHS Board†	Greater Glasgow and Clyde	18.42	p<0.001	0.34	p=0.025
	Lothian	26.86		0.37	
	Lanarkshire	11.93		0.27	
	Grampian	20.09		0.24	
	Tayside	14.11		0.23	
	Fife	14.61		0.22	
	Ayrshire and Arran	9.92		0.22	
	Highland	10.21		0.13	
	Forth Valley	14.25		0.26	
	Dumfries and Galloway	5.66		0.12	
	Borders	9.50		0.20	
	Western Isles	5.82		0.38	
	Shetland	6.33		0.00	
	Orkney	5.22		0.45	
Scottish Index of Multiple Deprivation	1 (most deprived)	10.02	p=0.007	0.22	p=0.310
	2	12.65		0.24	
	3	15.49		0.21	
	4	18.04		0.27	
	5 (least deprived)	25.95		0.35	
Month	May (17 May–6 June 2021)	4.40	p=0.002	0.04	p=0.003
	June (7 June–4 July 2021)	7.27		0.09	
	July (5 July–1 August 2021)	14.09		0.26	
	August (2 August–5 September 2021)	24.56		0.43	
	September (6–26 September 2021)	31.95		0.43	

* Test of difference in mean weekly travel events per 10 000 population.

† NHS Boards are ranked by population size from high-to-low.

Some age-and-sex-specific trends were also observed, with younger (20–29y) working-age females and older (30–39y and 50–59y) working-age males having the greatest travel frequencies (online supplemental file S5a). Furthermore, some destinations (Poland, Pakistan, Bulgaria and Romania) had relatively higher proportions of travellers resident in areas of high deprivation (low SIMD score) in Scotland (online supplemental file S5b). Despite temporal variation, some geographic consistency was observed, with more populous regions tending to have higher travel rates (online supplemental file S5c).

### Risks and population impact of SARS-CoV-2 importations through international travel during the period of the traffic light system

**Figure 3:**
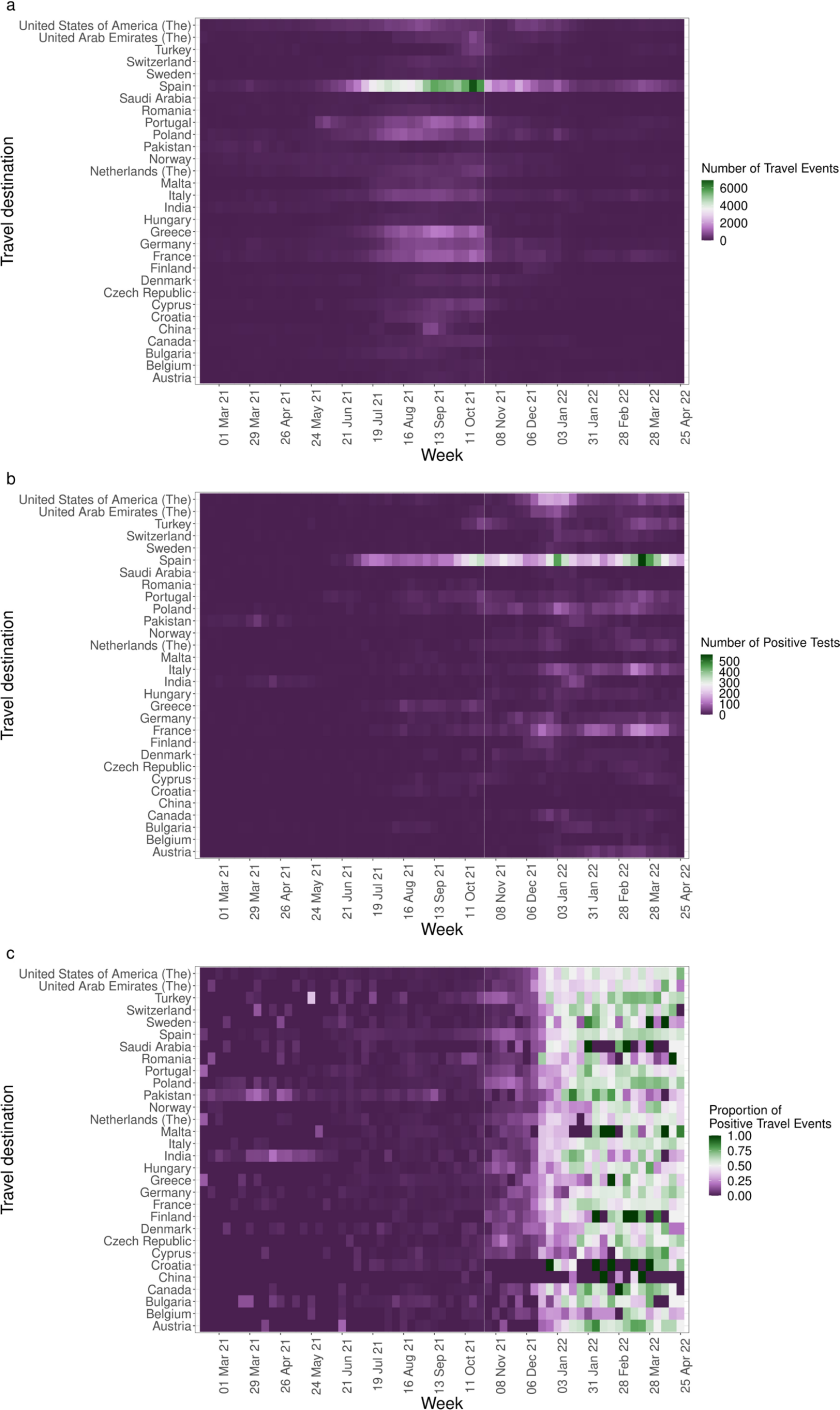
Patterns of international travel and associated SARS-CoV-2 infections in Scotland. Weekly (**a**) international travel events, (**b**) SARS-CoV-2 positive tests, and (**c**) proportions of SARS-CoV-2 positive travel events, spanning w/c 15 February 2021 to w/c 25 April 2022.

Figure 3 summarises weekly numbers of travel events and travel-related SARS-CoV-2 cases, among PCR-tested individuals, for each of the top 30 most frequently visited travel destinations. Overall, there was a 324% increase in SARS-CoV-2 cases, comparing the weeks with the highest travel frequency in the pretraffic light (w/c 5 April 2021) and traffic light (w/c 13 September 2021) periods. For some countries, periods of increasing case numbers appeared to coincide with increasing travel frequency. However, in other instances, increasing case numbers appeared decoupled from travel frequency and instead appeared to be explained by SARS-CoV-2 importation risk (the proportion of traveller PCR tests with a positive result).

**Figure 4:**
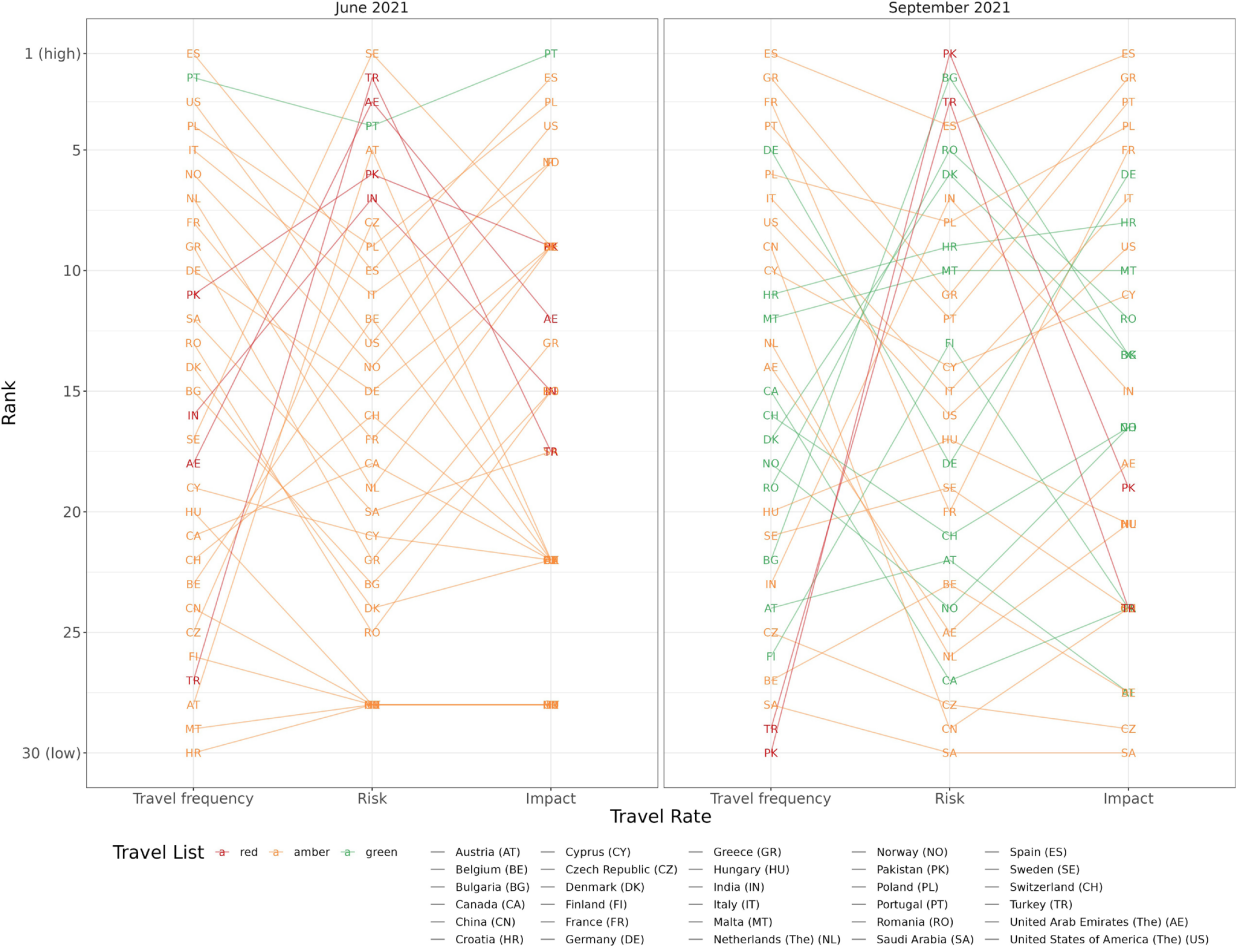
Top 30 most frequently visited international travel destinations for Scotland by risk and impact. Travel frequency, the number of travel events to the given destination; risk, infection importation risk (as measured by the proportion of PCR-confirmed SARS-CoV-2 cases among travel-related tests); impact, epidemiological impact (as measured by the proportion of Scottish cases of SARS-CoV-2 associated with international travel).

Figure 4 summarises the rankings of the top 30 most frequently visited countries according to travel frequency, SARS-CoV-2 importation risk and population impact (as measured by the proportion of all Scottish SARS-CoV-2 cases with an associated travel event). Countries ranking high across each metric represented the greatest risk when combining exposure and consequence. Notably, four red list countries appeared in the top seven, and two in the top three, ranking of SARS-CoV-2 importation risks during the lowest (June) and highest (September) travel frequency periods, respectively. However, one green list country had the highest combined ranking across all metrics during June, and by September, there was no discernible pattern of risk distinguishing green and amber list destinations.

During the period of the traffic light system, 3163 SARS-CoV-2 cases were detected through travel-based surveillance (5.8 per 10 000 Scottish population), compared with 342 778 cases detected through community-based surveillance (627.1 per 10 000 Scottish population) over the same period. Employing multivariable mixed-effects logistic regression, the odds of detecting SARS-CoV-2 cases increased from green-to-amber and amber-to-red list countries but was overall lower among cases identified through travel-based surveillance (travellers) than community-based surveillance (non-travellers). This finding was consistent regardless of RAG group, controlling for age group, sex, month and geographical location (see table 2, adjusted odd ratio, for details). Some variation in the effect of travel status was found according to age group, sex and month, however the relative odds of case detection were consistently greater among those with no recorded recent international travel event.

**Table 2:** Investigating risk factors for SARS-CoV-2 case detection during the period of the traffic light system (17 May 2021 to 30 September 2021) in Scotland

Factor	Level	Number (%) of positive test events (n=336 281)*	Number (%) of negative test events (n=1 647 991)*	Unadjusted OR (95% CI, p value)†	Adjusted OR (95% CI, p value)[Table-fn T2_FN3]
Travel status	No recent travel	333 206 (99.1)	1 457 766 (88.5)	Reference	Reference
	Travel to green list country	366 (0.1)	27 925 (1.7)	0.06 (0.05, 0.07; p<0.001)	0.05 (0.05, 0.06; p<0.001)
	Travel to amber list country	2583 (0.8)	156 578 (9.5)	0.07 (0.07, 0.08; p<0.001)	0.07 (0.07, 0.07; p<0.001)
	Travel to red list country	126 (0.04)	5722 (0.3)	0.10 (0.08, 0.12; p<0.001)	0.10 (0.09, 0.11; p<0.001)
Age group	20–39y	115 286 (34.3)	502 897 (30.5)	Reference	Reference
	0–19y	112 274 (33.4)	365 478 (22.2)	1.34 (1.33, 1.36; p<0.001)	1.21 (1.20, 1.22; p<0.001)
	40–59y	77 106 (22.9)	456 429 (27.7)	0.74 (0.73, 0.75; p<0.001)	0.73 (0.73, 0.74; p<0.001)
	60–79y	26 893 (8.0)	254 808 (15.5)	0.46 (0.46, 0.47; p<0.001)	0.46 (0.45, 0.46; p<0.001)
	80y+	4722 (1.4)	68 379 (4.1)	0.30 (0.29, 0.31; p<0.001)	0.28, (0.28, 0.29; p<0.001)
Sex	Female	168 826 (50.2)	904 078 (54.9)	Reference	Reference
	Male	167 455 (49.8)	743 913 (45.1)	1.21 (1.20, 1.21; p<0.001)	1.23 (1.22, 1.24; p<0.001)
Calendar month	September	123 647 (36.8)	557 606 (33.8)	Reference	Reference
	May	6751 (2.0)	101 607 (6.2)	0.30 (0.30, 0.31; p<0.001)	0.29 (0.28, 0.29; p<0.001)
	June	50 503 (15.0)	299 266 (18.2)	0.77 (0.76, 0.78; p<0.001)	0.70 (0.69, 0.71; p<0.001)
	July	58 949 (17.5)	261 172 (15.8)	1.02 (1.01, 1.03; p<0.001)	1.09 (1.08, 1.10; p<0.001)
	August	96 431 (28.7)	428 340 (26.0)	1.03 (1.02, 1.04; p<0.001)	1.06 (1.05, 1.07; p<0.001)
Deprivation (SIMD) score	5 (least deprived)	67 822 (20.2)	368 719 (22.4)	Reference	Reference
	1 (most deprived)	76 963 (22.9)	315 764 (19.2)	1.33 (1.31, 1.34; p<0.001)	1.13 (1.12, 1.14; p<0.001)
	2	69 334 (20.6)	313 912 (19.0)	1.20 (1.19, 1.21; p<0.001)	1.10 (1.09, 1.12; p<0.001)
	3	58 327 (17.3)	305 827 (18.6)	1.04 (1.02, 1.05; p<0.001)	1.03 (1.02, 1.05; p<0.001)
	4	63 845 (19.0)	343 769 (20.9)	1.01 (1.00, 1.02; p=0.114)	1.00 (0.99, 1.01; p=0.7)

* Numbers based on 1 984 272 SARS-CoV-2 test events with complete information across all variables.

† Crude odds ratioOR of SARS-CoV-2 case detection estimated from single-factor binary logistic regression models.

‡ Adjusted odds ratiosORs of SARS-CoV-2 case detection from multivariable binary logistic regression model (adjusted for age group, sex, month and SIMD) fitting a random effect to control for health board location.

SIMDScottish Index of Multiple Deprivation

Several demographic factors were identified as independently associated with the odds of detecting SARS-CoV-2, regardless of international travel status, with cases more likely detected among 0–19y relative to 20–39y age groups, among males relative to females, and among individuals’ resident in more deprived areas. These predictors of SARS-CoV-2 cases differed from that observed among travellers, as highlighted also by differences in the modelled predicted probabilities when comparing traveller and non-traveller groups (results not shown) and the patterns of travel frequency.

### Risks and population impact of SARS-CoV-2 novel variant importations through international travel during the period of the traffic light system

**Figure 5:**
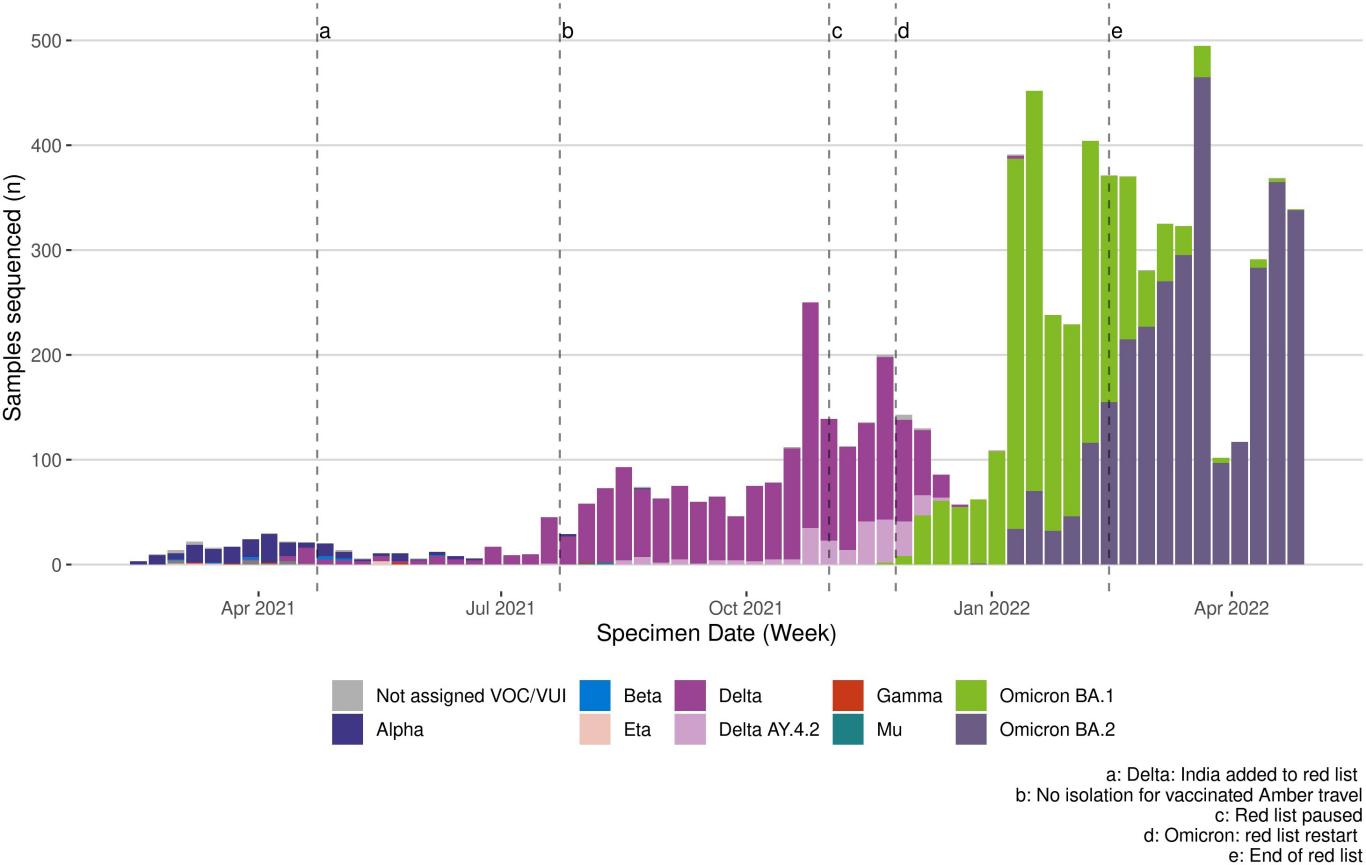
Weekly count of PCR-confirmed cases undergoing whole genome sequencing by SARS-CoV-2 variant type. VOC, variant of concern; VUI, variants of interest.

Figure 5 shows the weekly numbers of sequenced SARS-CoV-2 cases by variant group against a timeline of travel policy changes. For most travel destinations and periods of the epidemic, the most likely imported VOC reflected the dominant variant circulating in the UK representing 50% or more of sequenced cases (online supplemental file S6). However, some variation in the risk of VOC importation was observed across travel destinations, particularly during the period of Alpha variant dominance although based on small numbers of sequenced cases.

**Figure 6:**
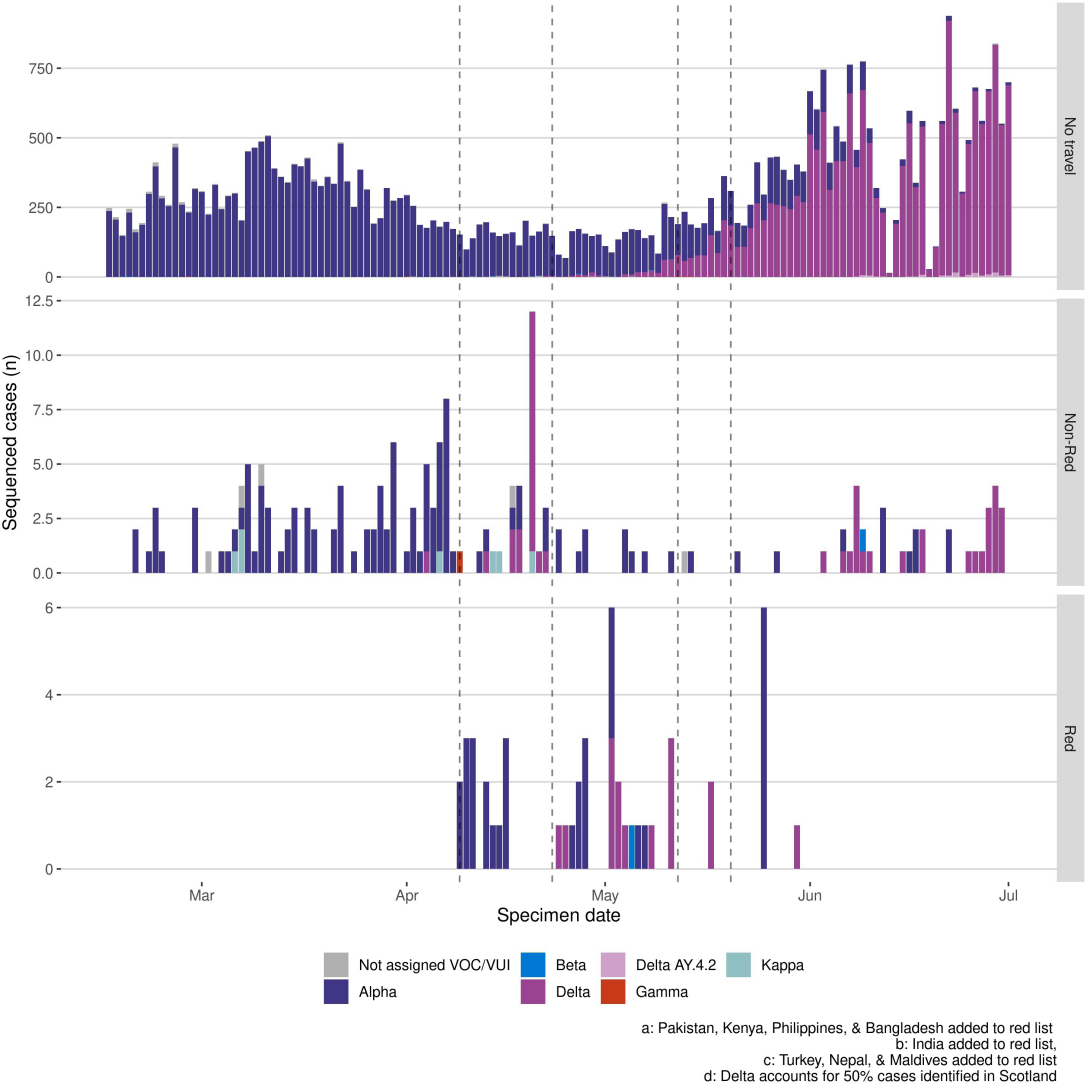
Weekly count of whole genome sequenced SARS-CoV-2 cases during period of Delta variant emergence. Numbers are shown among non-traveller community cases (top panel), travellers returning from non-red destinations (middle panel) and travellers returning from red-list travel destinations (bottom panel). VOC, variant of concern; VUI, variants of interest.

The Delta variant was identified in Scotland on 4 April 2021 before the relaxation of travel restrictions for some destinations through the traffic light system. Figure 6 compares weekly numbers of variants identified among sequenced SARS-CoV-2 cases returning from non-red and red-list travel destinations, alongside non-traveller community cases during the period in which Delta emerged. Although several countries were added to the red list from 9 April 2021, Delta was primarily detected among travellers returning from non-red list countries (except for a short period from late April to end of May). Community transmission was evident from late-April, following which Delta was relatively more frequently identified among non-travellers. Delta replaced Alpha to become the dominant variant in Scotland from 19 May 2021 (Figure S7, Supplementary Material).

**Figure 7:**
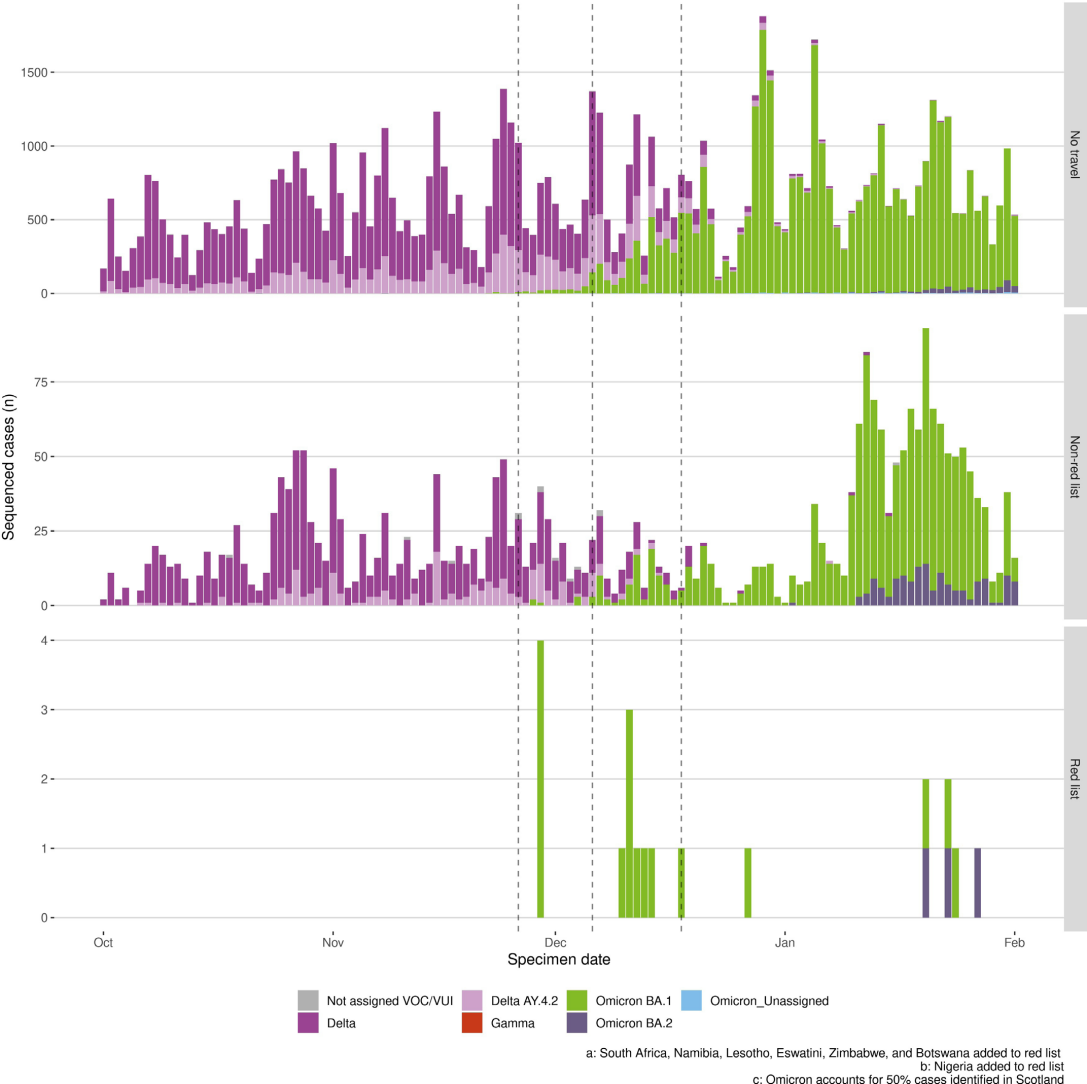
Weekly count of whole genome sequenced SARS-CoV-2 cases during period of Omicron variant emergence. Numbers are shown among non-traveller community cases (top panel), travellers returning from non-red destinations (middle panel) and travellers returning from red-list travel destinations (bottom panel). VOC, variant of concern; VUI, variants of interest.

Shortly after the traffic light system was removed on 4 October 2021, Omicron (BA.1 sublineage), emerged with the specimen of the first known case in Scotland dated 22 October 2021 (Figure S7, Supplementary Material). In contrast to Delta, Omicron was first identified in the community among non-travel-related cases (figure 7), with only 2.6% of sequenced Omicron cases associated with recent international travel compared with 22.1% of Delta cases in the first 4 weeks of their respective emergence. Overall, both Delta and Omicron were more frequently detected among non-travellers and similar to Delta, detections of Omicron were higher in those returning from non-red list than red list countries.

## Discussion

With the ongoing emergence of novel variants able to infect vaccinated and previously infected individuals, SARS-CoV-2 can be expected to persist globally with continued spread of novel variants of public health significance. While acquired immunity either from prior waves of infection or from vaccines are now mediating disease severity in most cases of infection, the future evolution of SARS-CoV-2 remains unpredictable. In the event of a new VOC emerging, border closures may provide a means for reducing the numbers of imported cases and control the progression of outbreaks locally. However, it is well recognised that unless implemented rapidly and informed by the true risks, the public health benefit is limited.

Once community transmission has been established, risk-based quarantine and post-travel screening may be proportionate measures to reduce the public health consequences of international travel. Risk-based screening measures were reintroduced by several nations, including the UK, owing to increasing global COVID-19 spread.^19^ However, there is a lack of understanding of travel policy effectiveness predating COVID-19, including for pandemic influenza and other emerging infectious diseases.^27 28^ Most studies assessing the public health effects of COVID-19 travel-related policies have used modelling approaches in the absence of empirical data, and have focused on the early phase of the pandemic when reduced international travel was of paramount interest to curb the global spread of disease.^2 20 21 29 30^ One study suggests that the quarantine of symptomatic COVID-19 cases is insufficient to significantly delay the onset of an epidemic.^31^

The traffic light system employed in the UK during 2021 aimed to provide a risk-based approach to targeting quarantine and screening measures for travellers returning from specific destinations. To the best of our knowledge, our study is the first to evaluate the potential public health effects of this measure. First, our study shows, as expected, that international travel increased substantially during the period of the traffic light system. The highest travel rates were seen for working-aged adults, those resident in more populous health boards, and in low deprivation areas. The ability to characterise patterns of travel may have implications for targeting safe travel public health messaging in the prevention of future VOC importations.

Second, travel was most frequent to amber list countries during the traffic light period despite the mandatory requirement for self-isolation. This finding was corroborated across PLF and CAA data sets despite known limitations, with the former involving self-submitted information and therefore reliant on compliance, and with the latter expected to have comparatively underrepresented travel from countries with few or no direct flights into Scotland but overrepresented travel from airports operating as central hubs.

Third, there were significant differences in the rates of SARS-CoV-2 infections detected among travellers across age groups and deprivation, but not for sex, aligning with patterns of travel frequency. However, cases of SARS-CoV-2 were more likely detected through community-based than travel-based surveillance overall during the traffic light period. Furthermore, although travel-related SARS-CoV-2 detections increased with decreasing deprivation, the odds of SARS-CoV-2 detections overall increased with deprivation while controlling for travel. Socioeconomic status is likely to play a role, with wealthier people more easily able to travel internationally, hence the greater SARS-CoV-2 detection rate among this group, but also protect themselves from infection thereby reducing their baseline infection risk. Overall, these findings highlight the complexity in understanding predictors of case importations and that patterns of travel may be a good proxy indicator.

The likelihood of detecting a SARS-CoV-2 infection among travellers increased from green-to-amber-to-red list countries, although with some variation when examining individual travel destinations. The highest frequency of travel was seen for an amber list country, resulting in relatively high numbers of imported SARS-CoV-2 cases when coupled with importation risk (proportion of travellers testing positive) together with a high population impact (proportion of national Scottish SARS-CoV-2 cases attributed to travel). However, our study shows that despite fewer travel events, the highest SARS-CoV-2 importation risk was associated with a green list country in June 2021. Furthermore, by September 2021, a number of green list countries ranked higher than red list countries for population impact, highlighting the complexity of proportionate applications of RAG systems. We note, however, that our study does not assess the impact of quarantine and isolation measures in place for those returning from red and amber list countries as there is no suitable control group. Quarantine and isolation measures are expected to have reduced the population impact of international travel. Travel frequency and the context of existing community circulation are key factors affecting the public health effects of international travel, along with the epidemiological situation of the travel destination. Identifying specific countries as high risk without considering the speed of spread of SARS-CoV-2 variants to other regions internationally, coupled with large differences in international surveillance efforts, will severely limit the effect of a traffic-light system.

Finally, our study spanned two novel SARS-CoV-2 variant introductions. Stringent travel restrictions did not prevent the introduction of Delta into the UK, while the risk-based red list policy did not prevent establishment in the community once introductions had occurred. However, decreased case numbers were observed for some red list countries during the Delta wave despite a high SARS-CoV-2 importation risk, likely owing to decreased travel to these destinations. The rapidity by which community transmission was established in the case of Omicron, with most initial cases detected among non-travellers, suggests that the direct impact of travel to red list countries during this period was likely minimal, especially given the requirement for quarantine. It was not possible however to assess the impact of the evolving epidemiological situation in red list countries and degree of global spread on the risks of Omicron variant importations from amber and green lists countries.

Our study has a number of limitations. First, laboratory surveillance data may not capture all travellers; an unknown number of individuals were exempt from testing and compliance is not quantifiable. Any potential self-reporting bias has not been addressed. While Scotland had the autonomy to implement its own travel policies during the study period, these aligned with the rest of the UK. There was unrestricted travel within the UK and individuals arriving elsewhere in the UK with onward travel to Scotland were not captured in traveller tests. Second, deductions of SARS-CoV-2 infection risks across travel-based and community-based surveillance groups must be made with caution. The greater odds of detecting SARS-CoV-2 cases through community surveillance may be explained by the targeted testing of symptomatic individuals and known close contacts of confirmed cases.

Furthermore, those entering the UK were required to take a predeparture test during the traffic light period, so the proportion of cases in this group is expected to reflect the risk of SARS-CoV-2 importation—combining the risks of a travel-associated infection and testing negative prior to departure. This should not preclude the validity of comparing SARS-CoV-2 case frequency over time, by travel destination and across demographic and geographic groups. Third, case misclassifications may have arisen, with some overascription of infections to the period of international travel (the acquisition of infection before or after travel cannot be ruled out, including from household transmission). Fourth, in the absence of a suitable control population or period, our study did not assess the reduction in SARS-CoV-2 case incidence in Scotland attributable to the traffic light system.

## Conclusions

Our findings show that risk-based postarrival screening undertaken in Scotland did not, in practice, prohibit the importation of SARS-CoV-2 cases, or the establishment of SARS-CoV-2 VOC in the Scottish community, arising through international travel. Overall, SARS-CoV-2 case importation risks did not strictly follow RAG designations. This is likely explained by travel frequency, regardless of country risk status, coupled with rapid global spread and local community transmission limiting the value of red lists to control VOC importations and population impact. Our study explores unique data on travel and SARS-CoV-2 testing and sequencing, and although no specific recommendations are made, it provides valuable learnings that may support resource allocation and decision making in Scotland during any future emergency response to COVID-19 or pandemics of other pathogens.

These lessons may be of relevance to other nations and infectious diseases although differences in the epidemiology, disease surveillance systems and travel policy implementations should be borne in mind. While it is clear that risk-based travel restrictions may have limited value in isolation in the case of a pathogen like SARS-CoV-2, which has the potential to spread rapidly, they could potentially work more effectively if international variant surveillance systems were compatible, and with information shared in a timely and open manner, to rapidly detect and respond to new variants as they arise. It should be noted that while Scotland has the autonomy to implement its own travel policies, the impact of unrestricted travel within the UK warrants specific investigation.

## supplementary material

10.1136/bmjopen-2024-085332 online supplemental file 1

## Data Availability

Data may be obtained from a third party and are not publicly available.
