# Supplementary material for: Evaluation of risk-based travel policy for the COVID-19 epidemic in Scotland: a population-based surveillance study
Source: BMJ Open. 2024 Nov 29;14(11):e085332. doi: 10.1136/bmjopen-2024-085332 (PMC11624746; doi:10.1136/bmjopen-2024-085332)
Supplement: online supplemental file 1 [file bmjopen-14-11-s001.pdf]

Evaluation of risk-based travel policy for the COVID-19 epidemic in Scotland: a population-based surveillance study

Supplementary Material

Authors: Isobel McLachlan[1,†], Selene Huntley[1,†], Kirstin Leslie[1,†], Jennifer Bishop[1], Christopher Redman[1], Gonzalo Yebra[1], Sharif Shaaban[1], Nicolaos Christofidis[1], Samantha Lycett[2], Matthew T.G. Holden[1,3], David L. Robertson[1,4], Alison Smith-Palmer[1,], Joseph Hughes[1,4], Sema Nickbakhsh[1,5,*]

Affiliations
[1]Public Health Scotland, Gyle Square, 1 South Gyle Crescent, Edinburgh EH12 9EB, United Kingdom
[2]Roslin Institute, Easter Bush Campus, University of Edinburgh, Edinburgh, EH25 9RG, United Kingdom
[3]School of Medicine, University of St Andrews, North Haugh, St Andrews, KY16 9TF, United Kingdom
[4]MRC-University of Glasgow Centre for Virus Research, 464 Bearsden Road, Glasgow, G61 1QH, United Kingdom
[5]School of Biodiversity, One Health & Veterinary Medicine, Garscube Campus, University of Glasgow, Glasgow, G61 1QH, United Kingdom
[†]Shared first authors

*Corresponding authors: Dr. Sema Nickbakhsh (sema.nickbakhsh@phs.scot)

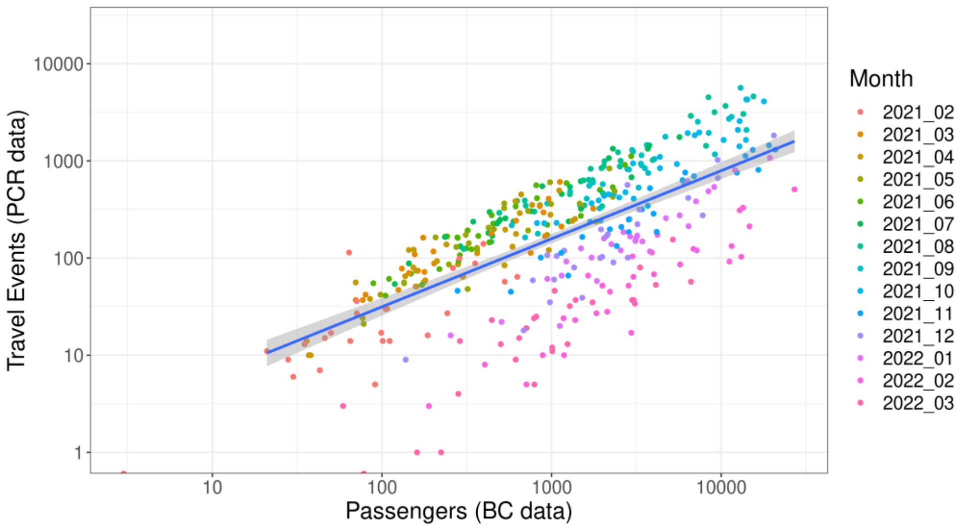

Figure S1. Assessing correlation between data sources informing on international travel frequency. Weekly numbers of passengers arriving into Scotland (based on Border Control Passenger Locator Forms) and international travel events (based on COVID-19 PCR-test surveillance data).

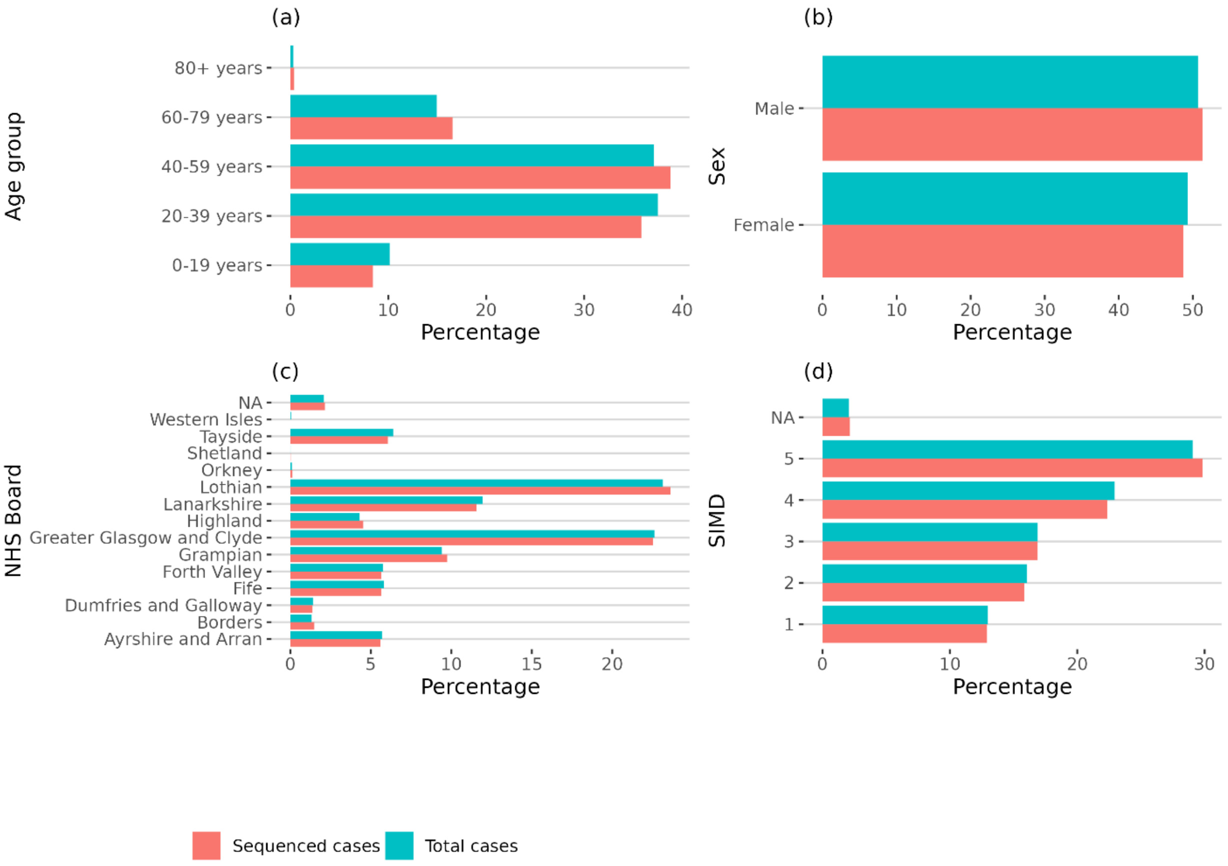

Figure S2. Demographic and geographic distributions of total COVID-19 cases and sequenced COVID-19 cases in Scotland. Distributions are shown by (a) age, (b) sex, (c) NHS Board of residence, and (d) Scottish Multiple Index of Deprivation (SIMD) of NHS Board residence; an increasing score reflects decreasing deprivation. NA refers to laboratory records that were not linkable to the factor of interest by postcode.

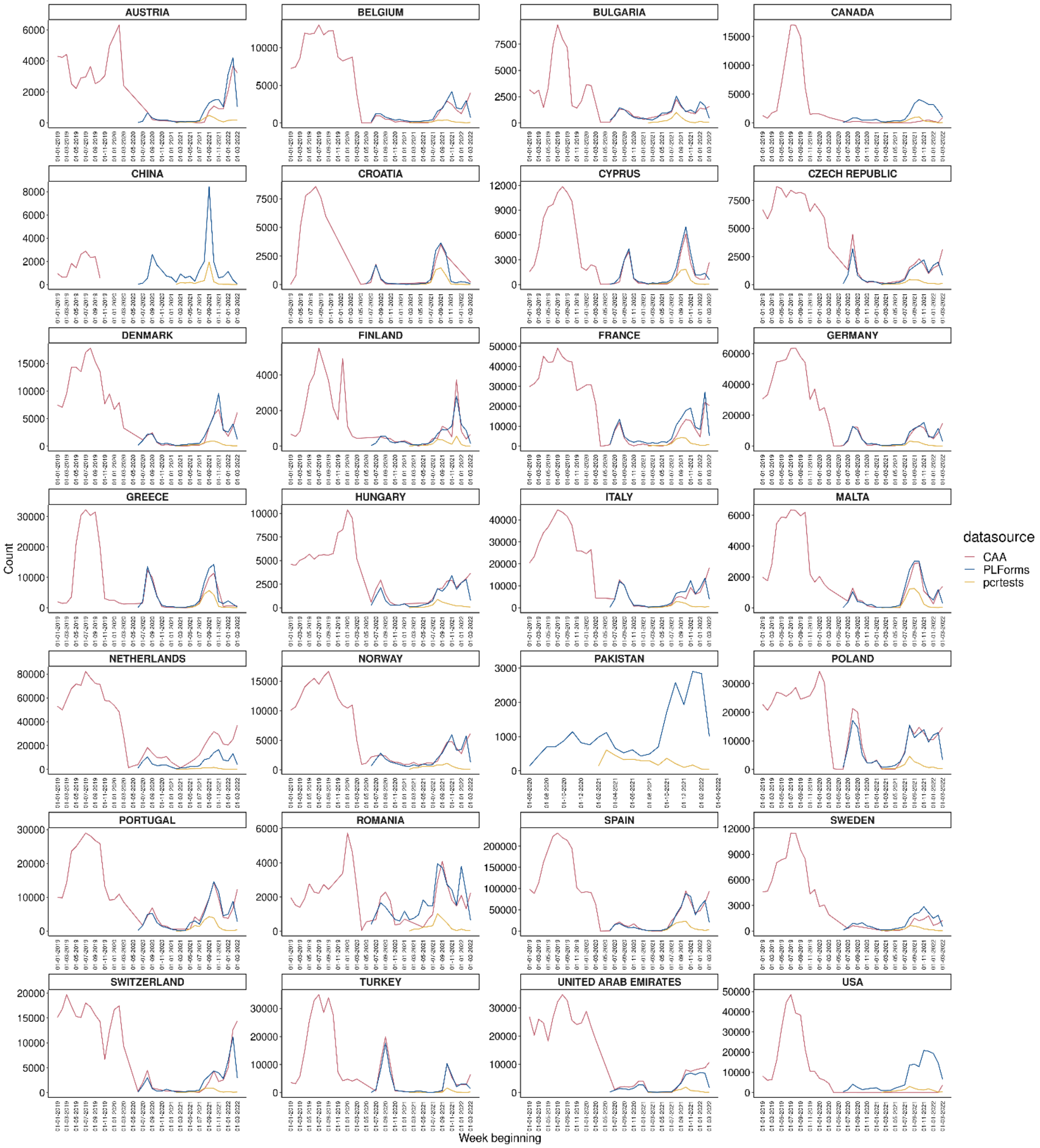

Figure S3. **Comparing longitudinal trends in weekly frequency of international travel by Scottish residents across data sources.** January 2019 to March 2022. BC = Border Control (Passenger Locator Forms); CAA = Civil Aviation Authority; PCR test = SARS-CoV-2 tested individuals with a recent international travel event.

(a)

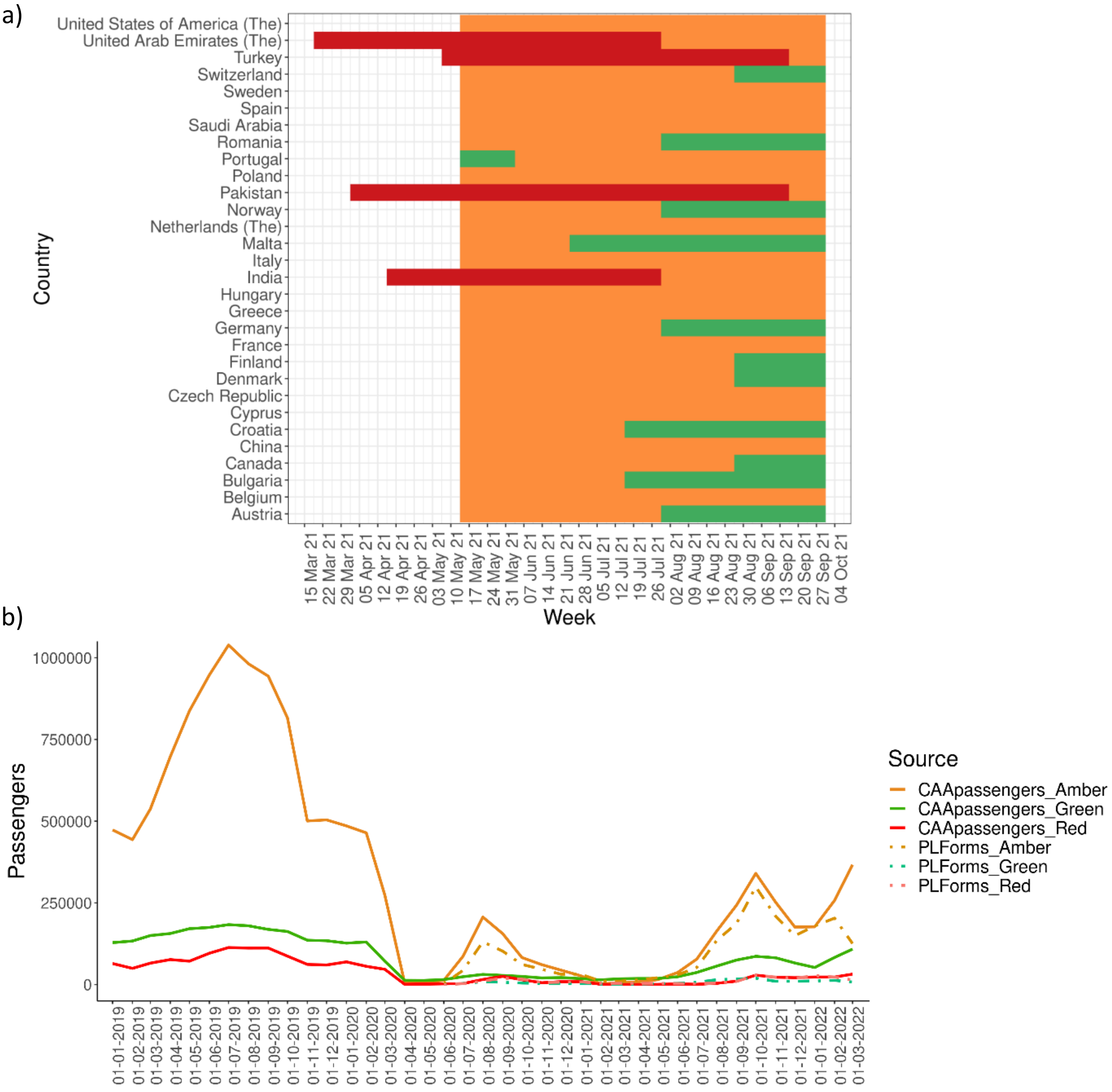

Figure S4. Assessing the impact of the traffic light system on travel frequency in Scotland. (a) Summary of Red-Amber-Green list designations in each week of the traffic light period shown for the top 30 most frequently visited countries (as determined based on COVID-19 PCR-tested Scottish residents). (b) Weekly numbers of passengers into Scotland retrospectively applying Red-Amber-Green (RAG) group classifications from the traffic light period to the travel destinations in Civil Aviation Authority and Passenger Locator Form datasets, to compare periods spanning pre-and-post introduction of the traffic light system.

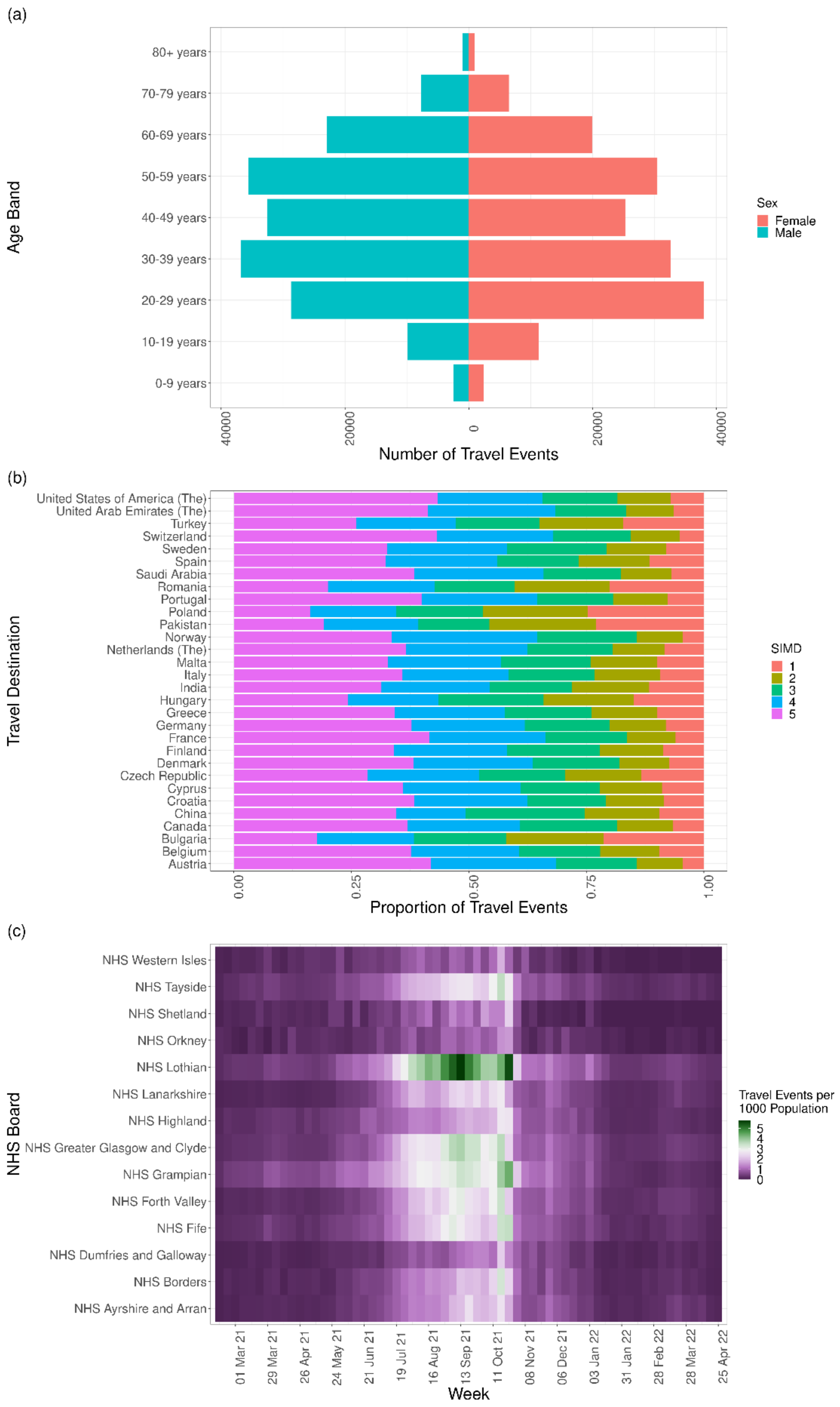

Figure S5. Demographic and geographic distributions of Scottish residents with an international travel even within 14-days of requesting a COVID-19 PCR-test, w/c 15th February 2021 to w/c 24th April 2022. Distributions are shown by (a) age-sex, (b) Scottish Multiple Index of Deprivation; SIMD (an increasing score reflects decreasing deprivation), and (c) by NHS Board of residence.

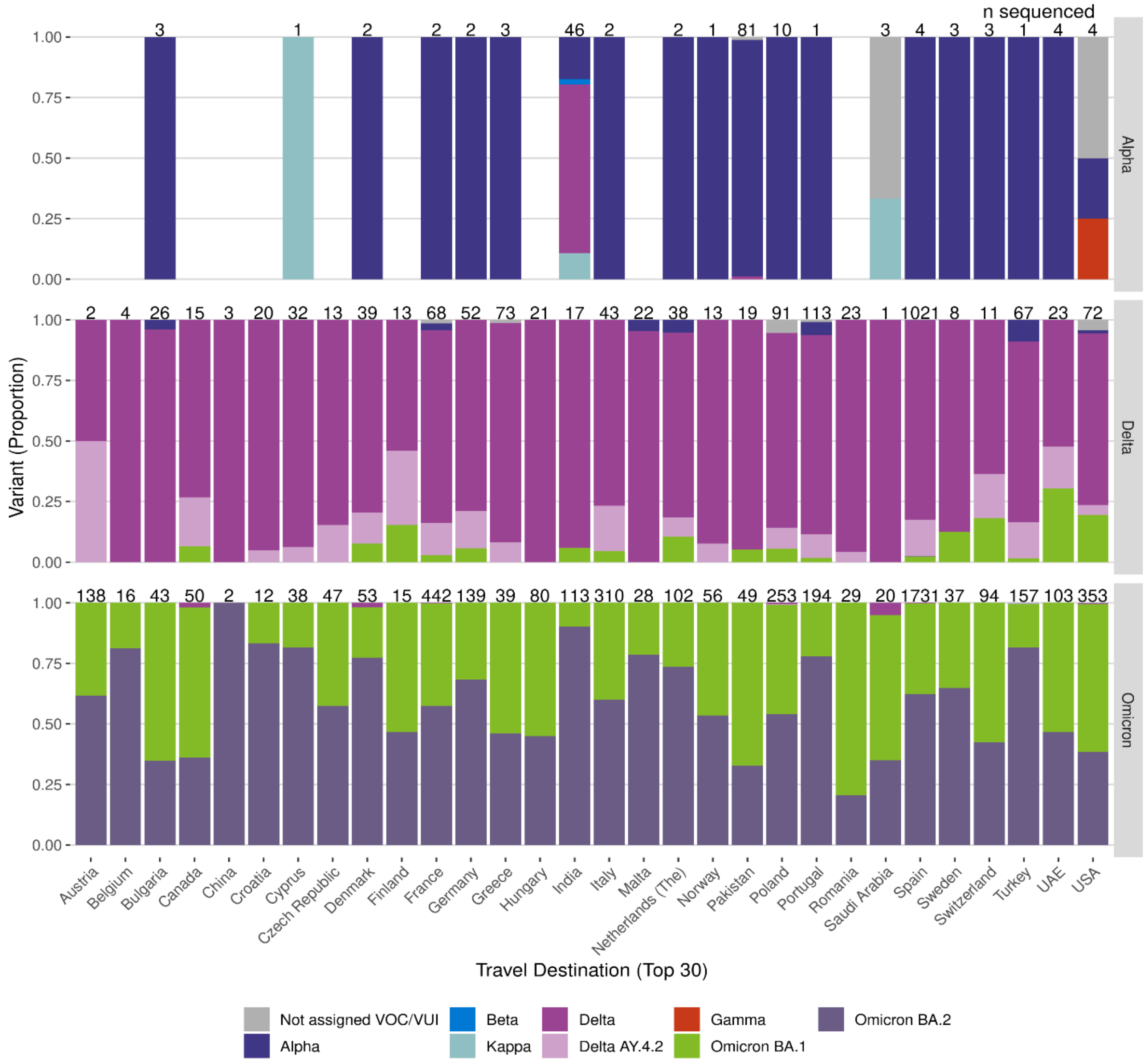

Figure S6. Proportion of PCR-confirmed SARS-CoV-2 cases who had undergone whole genome sequencing and classified infected with a SARS-CoV-2 Variant of Concern. Data are shown from travellers to top-30 destinations during periods of Alpha, Delta, and Omicron dominance.

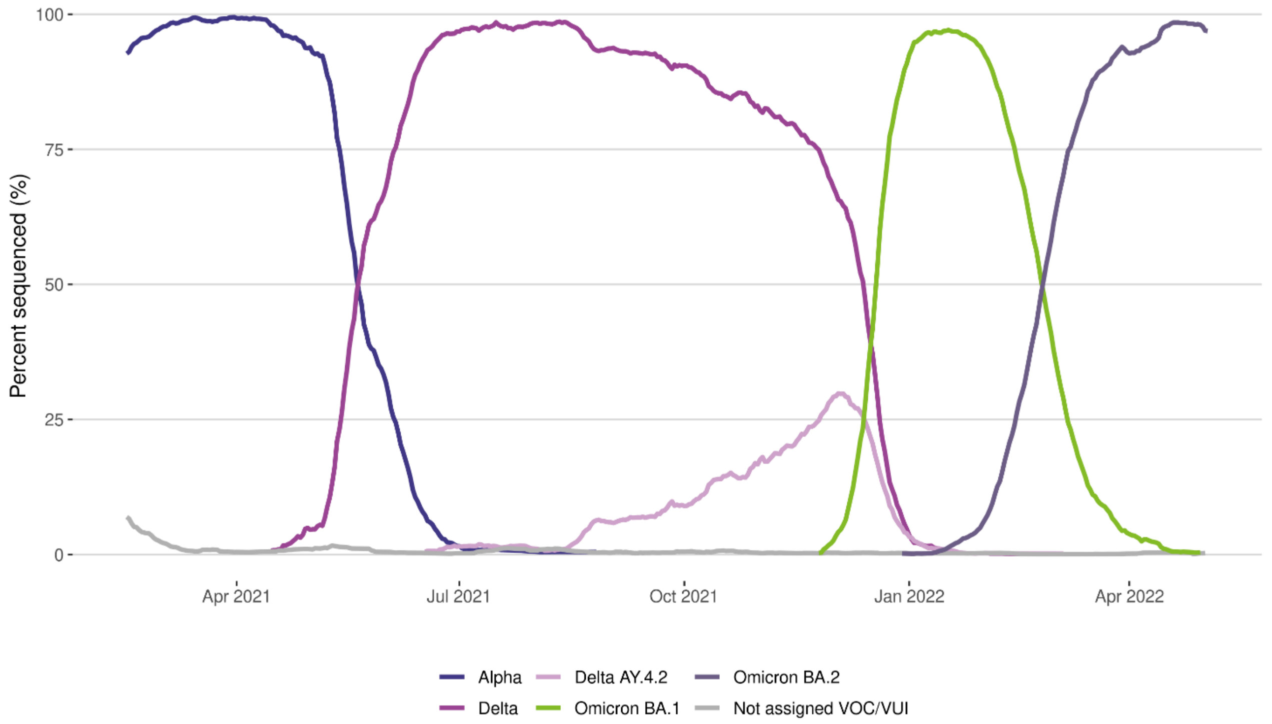

Figure S7. Epidemic curve of dominant SARS-CoV-2 Variants of Concern identified by whole genome sequencing in the Scottish population during the study period (15th February 2021 to 3rd May 2022).
