# Supplementary material for: Evaluation of risk-based travel policy for the COVID-19 epidemic in Scotland: a population-based surveillance study
Source: BMJ Open. 2024 Nov 29;14(11):e085332. doi: 10.1136/bmjopen-2024-085332 (PMC11624746; doi:10.1136/bmjopen-2024-085332)
Supplement: Supplementary file 2 [file bmjopen-2024-085332.reviewer_comments.pdf]

PEER REVIEW HISTORY

BMJ Open publishes all reviews undertaken for accepted manuscripts. Reviewers are asked to complete a checklist review form and are provided with free text boxes to elaborate on their assessment. These free text comments are reproduced below.

ARTICLE DETAILS

Title (Provisional)

Evaluation of risk-based travel policy for the COVID-19 epidemic in Scotland: A population-based surveillance study

Authors

McLachlan, Isobel; Huntley, Selene; Leslie, Kirstin; Bishop, Jennifer; Redman, Christopher; Yebra, Gonzalo; Shaaban, Sharif; Christofidis, Nicolaos; Lycett, Samantha; Holden, Matthew T.G.; Robertson, David L; Smith-Palmer, Alison; Hughes, Joseph; Nickbakhsh, Sema

---

VERSION 1 - REVIEW

**Reviewer**	**1**
**Name**	**Toshkov, Dimiter**
**Affiliation**	**Leiden University**
**Date**	**08-Mar-2024**
**COI**	**No competing interests.**

This manuscript intends to study the effects of travel restrictions on the frequency of travel and indicators of the COVID-19 pandemic, such as the SARS-CoV-2 case importations, cases and variants.

While the impact of border restrictions on the spread of the COVID-19 virus is a relevant research question, I do not find that the current manuscript provides a clear and convincing analysis that sheds new light on this question. There is a lot of descriptive data provided in the paper, but not all comparisons are presented in an informative way. Most importantly, the central result that people who travelled did not have a higher change to test positive then the rest of the population has unclear interpretation and significance. (cf. page 13: ‘Overall, the rate of SARS-CoV-2 case detection was estimated to be 17 per 1,000 among those with an international travel event compared to 190 per 1,000 among those without an international travel event over the same period.’)

Simply put, this difference could be driven by selection into testing by the general population based on early symptoms (while testing was compulsory for all travelers) and is subject to reverse causality in which people who test (or felt symptoms before getting tested) would not

have travelled. Therefore, I find it impossible to draw any implications from the data for policy or for understanding the impact of travel restrictions on the trajectory of the pandemic.

More generally, while the title of the paper references ‘evaluation’ and ‘effects’, the descriptive nature of the study, coupled with the lack of attention to causal modeling, allows for no statements about (causal) evaluation and effects.

I also find it confusing that the frequency of travel during the period with ‘traffic light’ system of travel advice is compared to the period immediately before which had stronger restrictions than to a period with no restrictions. (page 13: ‘The frequency of international arrivals 217 into Scottish airports subsequently increased by 754% from May 2021 to September 2021, compared with a 12% increase over 218 the same period in 2019 (Figure 1).’) Even if there was a bigger increase from May to September 2021, the *frequency* of travel in September 2021 could still have been much lower than in September 2019, and this is the more relevant information for assessing the impact of policy on travel.

Reviewer 2

Name Meyerowitz-Katz, Gideon

Affiliation University of Wollongong Faculty of Health and Behavioural Sciences, School of Nursing

Date 15-Mar-2024

COI I have no competing interests to declare

The authors have conducted a largely descriptive review of the importation of COVID-19 cases into Scotland during the first two years of the pandemic. In general, the paper is well-written and makes for interesting reading.

My main concern with the paper as written is that it does not acknowledge the primary substantial limitation in conducting a review of COVID-19 importation into Scotland, which is that travel is entirely unrestricted within the UK. Moreover, there was (and is) a complex border relationship between the EU and UK in Northern Ireland. It is therefore challenging to infer specifics about the risk of imported cases looking only at Scottish data, as clearly international travel of Scottish residents is not the only method through which new cases can be imported.

This limitation is also important to consider as part of the discussion of this paper. I am not certain it makes sense to review the international travel policies of Scotland alone, nor discuss border controls. Unless Scotland breaks from the UK, there will never be a hard border that can be closed between Newcastle and Edinburgh, which limits the ability for international travel to prevent importation.

I think that this limitation requires more discussion by the authors. It is possible that the higher rates of transmission in the general population are due to importations from elsewhere in the UK, for example.

The other limitation that the authors have not adequately engaged with is the issue that they mention in their limitations section briefly - that testing differences between travelers and the general population likely explain a large portion of any differences. During the time-period analyzed, the travelers had around 1,300 tests per 1,000, of which roughly 1.5% were positive. In the UK as a whole in the same time period, the test-positivity rate varied between 0.2-13%. Clearly, testing for travel and testing for the general population are dramatically different, and thus any inferences made on the basis of these tests must be taken with extreme care. It might be useful, for example, to adjust the logistic regression with a term for % test positivity as a marker of how many tests per case were being done in each different situation.

This being said, I do think the paper makes a useful point, which is that local policy during COVID-19 was not always based on a properly calculated risk. The fact that, even given the limitations, there were likely fewer cases among travelers than in the general population calls into question whether the system was the best allocation of resources at the time. This does also raise the question of socioeconomics, however, because it is likely that in part the reason that cases were relatively sparse among international travelers during the pandemic has a lot to do with the ability of wealthier people to both protect themselves from disease and travel internationally.

VERSION 1 - AUTHOR RESPONSE

Reviewer: 1

Dr. Dimiter Toshkov, Leiden University Comments to the Author:

This manuscript intends to study the effects of travel restrictions on the frequency of travel and indicators of the COVID-19 pandemic, such as the SARS-CoV-2 case importations, cases and variants.

While the impact of border restrictions on the spread of the COVID-19 virus is a relevant research question, I do not find that the current manuscript provides a clear and convincing analysis that sheds new light on this question. There is a lot of descriptive data provided in the paper, but not all comparisons are presented in an informative way. Most importantly, the central result that people who travelled did not have a higher change to test positive then the rest of the population has unclear interpretation and significance. (cf. page 13: ‘Overall, the rate of SARS-CoV-2 case detection was estimated to be 17 per 1,000 among those with an international travel event compared to 190 per 1,000 among those without an international travel event over the same period.’)

We thank the reviewer for their helpful comments. We believe our extensive descriptive data presents a novel provision of data-driven evidence around the impact of travel restrictions on

infection importation risks. We have reworded some of the statements around the comparisons so that the messages are clearer and provided some detail to make these comparisons more informative.

The analysis was made possible through the combination of the unique testing programmes in place during the COVID-19 pandemic and the healthcare data linkage capabilities in Scotland. We are not aware of alternative healthcare data sources available for addressing these questions.

We did not intend for the difference in case detections between travellers and non-travellers to imply differences in infection acquisition risk (as discussed in the manuscript). We have amended the text to make clearer the difference in case detection among travellers and non- travellers is with respect to travel versus non-travel-based surveillance systems (**see abstract lines 38-41, Methods lines 235-237, Results lines 354-361**). Our results also highlight the complexity of predicting case detection specific to travellers, and the overall risk from RAG designated countries, owing to multiple contributing factors.

Simply put, this difference could be driven by selection into testing by the general population based on early symptoms (while testing was compulsory for all travelers) and is subject to reverse causality in which people who test (or felt symptoms before getting tested) would not have travelled.

We agree with the reviewer. The discussion acknowledges that the 'targeted testing of symptomatic individuals and known close contacts of confirmed cases' (**lines 493-497**) in the non-traveller group may explain the greater odds of detecting SARS-CoV-2 cases compared to the traveller group. Additionally, the differences in testing between the general population (symptomatic, close contacts of cases and workplace testing) and travellers (post arrival tests for all international travellers) are outlined in the methods. See also response above – we have now made clearer that the comparison is with respect to detections by the comparative (travel and non-travel-based) surveillance systems.

Therefore, I find it impossible to draw any implications from the data for policy or for understanding the impact of travel restrictions on the trajectory of the pandemic.

We have demonstrated variation in the frequency of international travel-related cases across demographic factors and according to the travel destination, and that this did not always align with RAG designation. We have also shown risk-based post arrival screening did not prohibit importation of SARS-CoV-2 from international travel. We conclude that travel frequency along with rapid global spread and community transmission likely limited impact of red list designation. Given the uniqueness of the dataset and paucity of data-driven evidence in this area, we believe our findings will be of interest to public health and policymakers.

More generally, while the title of the paper references 'evaluation' and 'effects', the descriptive nature of the study, coupled with the lack of attention to causal modeling, allows for no statements about (causal) evaluation and effects.

The title has been amended to reflect that we have not made causal statements about the link between the travel policy and COVID-19 cases in Scotland.

I also find it confusing that the frequency of travel during the period with ‘traffic light’ system of travel advice is compared to the period immediately before which had stronger restrictions than to a period with no restrictions. (page 13: ‘The frequency of international arrivals 217 into Scottish airports subsequently increased by 754% from May 2021 to September 2021, compared with a 12% increase over 218 the same period in 2019 (Figure 1).’) Even if there was a bigger increase from May to September 2021, the *frequency* of travel in September 2021 could still have been much lower than in September 2019, and this is the more relevant information for assessing the impact of policy on travel.

This statement is included to illustrate that the increase in travel that was observed following the easing (but not lifting) of restrictions was greater than seasonal variation that was observed pre-pandemic. We also include comparison to the pre-pandemic period; during the traffic light period there was an 87.4% reduction in international flight passengers compared to the same period in 2019 (**line 284-286**) and most frequently visited destinations are largely consistent prior to and during the COVID-19 pandemic (lines **287-289** and figure S2).

Reviewer: 2

Dr. Gideon Meyerowitz-Katz, University of Wollongong Faculty of Health and Behavioural Sciences

Comments to the Author:

The authors have conducted a largely descriptive review of the importation of COVID-19 cases into Scotland during the first two years of the pandemic. In general, the paper is well- written and makes for interesting reading.

My main concern with the paper as written is that it does not acknowledge the primary substantial limitation in conducting a review of COVID-19 importation into Scotland, which is that travel is entirely unrestricted within the UK. Moreover, there was (and is) a complex border relationship between the EU and UK in Northern Ireland. It is therefore challenging to infer specifics about the risk of imported cases looking only at Scottish data, as clearly international travel of Scottish residents is not the only method through which new cases can be imported. This limitation is also important to consider as part of the discussion of this paper. I am not certain it makes sense to review the international travel policies of Scotland alone, nor discuss border controls. Unless Scotland breaks from the UK, there will never be a hard border that can be closed between Newcastle and Edinburgh, which limits the ability for international travel to prevent importation. I think that this limitation requires more discussion by the authors. It is possible that the higher rates of transmission in the general population are due to importations from elsewhere in the UK, for example.

We thank the reviewer for their constructive comments and agree that the unrestricted travel between Scotland and the rest of the UK presents a substantial contextual factor in understanding COVID-19 importation dynamics. Our research focused on examining COVID-19 importation directly into Scotland and while we acknowledge the significance of assessing the risk of importation from the rest of the UK, this falls out with the scope of our

study objectives and available datasets. Scotland has the autonomy to implement its own travel policy however during the study period the policy aligned with the rest of the UK. We have added further discussion to address the connection with the rest of the UK (**line 490- 493**).

The other limitation that the authors have not adequately engaged with is the issue that they mention in their limitations section briefly - that testing differences between travelers and the general population likely explain a large portion of any differences. During the time-period analyzed, the travelers had around 1,300 tests per 1,000, of which roughly 1.5% were positive. In the UK as a whole in the same time period, the test-positivity rate varied between 0.2-13%. Clearly, testing for travel and testing for the general population are dramatically different, and thus any inferences made on the basis of these tests must be taken with extreme care. It might be useful, for example, to adjust the logistic regression with a term

for % test positivity as a marker of how many tests per case were being done in each different situation.

We describe in the methods section the testing reasons for testing (**lines 157-160**) and have acknowledged this as a limitation in our discussion and in particular that caution should be made when making deductions about infection risk between traveller and non-traveller groups. We have also now made clearer that the comparison of case detections is being made with respect to the travel vs non-travel-based surveillance systems. We have considered the suggestion to include a test positivity term in the logistic regression however do not feel it would be appropriate due to the correlation between test positivity and our outcome variable, the odds of case detection. The result of increased odds of case detection from green to amber and amber to red list countries is also an important result.

This being said, I do think the paper makes a useful point, which is that local policy during COVID-19 was not always based on a properly calculated risk. The fact that, even given the limitations, there were likely fewer cases among travelers than in the general population calls into question whether the system was the best allocation of resources at the time. This does also raise the question of socioeconomics, however, because it is likely that in part the reason that cases were relatively sparse among international travelers during the pandemic has a lot to do with the ability of wealthier people to both protect themselves from disease and travel internationally.

We thank the reviewer and agree this is an important point and have added additional text to our discussion to make this clearer (**line 447-453**).

Reviewer: 1

Competing interests of Reviewer: No competing interests.

Reviewer: 2

Competing interests of Reviewer: I have no competing interests to declare

Please note we have also made some minor editorial changes throughout for clarity and readability particularly in relation to the structure of the methods. We have also subsequently

identified a data processing issue in relation to the logistic regression analyses which has led to some very minor changes and Table 2 has been updated accordingly.

VERSION 2 - REVIEW

**Reviewer**	**3**
**Name**	**Kelson, Zoe**
**Affiliation**	**University of Exeter, Mathematics**
**Date**	**20-Sep-2024**
**COI**	**None**

This population-based surveillance study aimed to assess the effects of risk-based travel restrictions on (i) international travel frequency, (ii) SARS-CoV-2 case importation risk, (iii) national SARS-CoV-2 incidence, and (iv) importation of SARS-CoV-2 variants in Scotland.

Reviewer comments:

"Data on the monthly number of passenger arrivals into Scottish airports, by country and airport of origin, were provided by the Civil Aviation Authority (CAA)"

and

"From June 2020, measures were introduced requiring all UK arrivals to complete a Passenger Locator Form (PLF) to support compliance with COVID-19 travel measures (23). The dataset contained weekly data for the period 28th June 2020 to 19th March 2022 on the number of PLFs submitted to Border Control and the originating countries from which passengers had travelled into Scotland"

The authors have made use of valuable data in an attempt to understand travel patterns.

"To enable like-for-like country-level comparison with PCR data, data on the Canary Islands and Madeira were merged with that of Spain and Portugal respectively in both the CAA and the PLFs. "

Can the authors please confirm that RAG status was consistently the same for these countries?

"Data on SARS-CoV-2 infections in Scotland were accessed from the NHS Scotland Corporate Data Warehouse (CDW). The dataset captured demographics (age, sex, postcode location) and test information (date of sample and test result). Reasons for testing included diagnostic confirmation for those with symptoms, asymptomatic testing of close contacts to support self isolation, workplace testing as part of the NHS Test & Protect system, and post arrival testing of travellers returning from international destinations. "

Can the authors please comment on whether this self-sampled cohort can be considered to be representative of cases across Scotland?

Can the authors please additionally consider using sample weights and modelling, based on age, sex, postcode, and various risks of exposure, to estimate scenarios of unknown cases in the (not-sampled) population across the country?

"When booking a post arrival COVID-19 test, individuals who had travelled internationally within the preceding 14 days were required to self-report the main country they had travelled to"

Can the authors please further discuss the potential for self-reporting bias?

"Monthly PCR test frequencies for travellers were strongly correlated with numbers of passengers into Scotland based on PLFs (Figure S1, Supplementary Material)."

and

"Although post-arrival tests were a mandatory travel policy, some exceptions were in place and compliance was not quantified (23). PLF data were therefore used to assess how representative the PCR test data was of travel frequency by destination during the traffic light period."

The authors do well to explore this.

"During the study period there were 317,570 samples from COVID-19 cases resident in Scotland that underwent SARS-CoV-2 whole genome sequencing. Samples which had a low-quality genome and samples that could not be linked to a positive SARS CoV-2 PCR test, and therefore could not be confirmed as a Scottish sample, were excluded (n=78)."

Can this cohort be considered to be representative (i.e. are case samples that underwent WGS similar to samples that did not)?

"Groups at risk of importing SARS-CoV-2 through international travel were also assessed by comparing travel frequencies across demographic (age, sex, relative deprivation of residential location, as measured by the Scottish Index of Multiple Deprivation; SIMD) and geographical (territorial NHS Board) factors. "

Insightful subgroup analyses have been explored.

"An ANOVA test was used to assess differences in mean travel frequencies across demographic and geographic groups, with a p-value <0.05 applied to indicate statistical significance."

Can the authors please confirm how normality was verified for the application of parametric statistical techniques?

"The potential impact of travel on the epidemiological situation in Scotland was assessed. To do so, numbers of travel-related SARS-CoV-2 cases were quantified as a proportion of all observed community cases of SARS-CoV-2 in Scotland"

Can uncertainty please be quantified and included in this assessment?

"The association between travel-based versus community-based surveillance and SARS-CoV-2 case detections was assessed in a test-negative case-control design for the traffic light system period (17th May 2021 to 30th September 2021)."

and

"Unadjusted odds ratios were initially quantified in univariate binary logistic regression models examining associations between SARS-CoV-2 infection and travel status, age group (0-19y, 20-39y, 40-59y, 60-79y, 80y+), sex (male versus female), month (May, June, July, August, September) and NHS Board (fourteen territorial health boards). Multivariable mixed-effects logistic regression modelling was then used to quantify the relative odds of SARS-CoV-2 case detection adjusting for age group, sex, and calendar month as fixed effects and NHS Board location as a random effect. Statistical interactions were assessed for all factors that were significant in the final multivariable model, with a p-value less than 0.05 indicating statistical significance."

and

"The glm function was applied for single effect statistical models and the lme4 (1.1-27.1 package) for mixed-effect models"

Appropriate modelling methods have been applied.

"Table 2. Investigating risk factors for SARS-CoV-2 infection during the period of the traffic light system (17th May 2021 to 30th September 2021) in Scotland."

Can the authors please clarify the outcome variable in these models (i.e. OR for what)?

How to interpret this table is currently unclear, can the authors please provide further clarification?

"Our study has a number of limitations. Firstly, laboratory surveillance data may not capture all travellers; an unknown number of individuals were exempt from testing and compliance is not quantifiable. While Scotland had the autonomy to implement its own travel policies during the study period these aligned with the rest of the UK. There was unrestricted travel within the UK and individuals arriving elsewhere in the UK with onward travel to Scotland were not captured in traveller tests. Secondly, deductions of SARS-CoV-2 infection risks across travel-based and community-based surveillance groups must be made with caution. The greater odds of detecting SARS CoV-2 cases through community surveillance may be explained by the targeted testing of symptomatic individuals and known close contacts of confirmed cases.

Furthermore, those entering the UK were required to take a pre-departure test during the traffic light period, so the proportion of cases in this group is expected to reflect the risk of SARS-CoV-2 importation - combining the risks of a travel-associated infection and testing negative prior to departure. This should not preclude the validity of comparing SARS-CoV-2 case frequency over time, by travel destination, and across demographic and geographic

groups. Thirdly, case misclassifications may have arisen, with some over-ascription of infections to the period of international travel (the acquisition of infection before or after travel cannot be ruled out, including from household transmission). Fourthly, in the absence of a suitable control population or period, our study did not assess the reduction in SARS-CoV-2 case incidence in Scotland attributable to the traffic light system."

A discussion on the study limitations has been provided by the authors.

Can the authors please do more to model uncertainties in an attempt to account for potential biases in the data and estimate the number of unknown cases under different scenarios and assumptions?

"Our findings suggest that country-specific post-arrival screening undertaken in Scotland did not prohibit the public health impact of COVID-19 in Scotland."

and

"Our findings show that risk-based post-arrival screening undertaken in Scotland did not, in practice, prohibit the importation of SARS-CoV-2 cases, or the establishment of SARS-CoV-2 VOC in the Scottish community, arising through international travel."

Of note, the aim of travel restrictions, the RAG system, and post-arrival screening, might not have been to prohibit international risks, but instead to reduce these risks to a level where health systems were better able to manage the speed of spread and volume of cases.

For a more nuanced evaluation, can the authors please consider comparing the outcomes to other hypothetical scenarios (e.g. what might be expected if travel was permitted without the RAG system and post-arrival screening in place, or a different suggestion for travel restriction and monitoring was introduced, or alternatively no travel was permitted at all)?

"Travel rates likely contributed to patterns of high SARS-CoV-2 case importation and population impact. "

Can the authors please re-consider whether this conclusion can be inferred from the scope of this study?

How are 'high' and 'impact' defined here?

Thanks for providing a copy of the STROBE checklist.

VERSION 2 - AUTHOR RESPONSE

We thank the reviewer for their considered review of our manuscript. We believe we have thoroughly addressed all the points raised. Please find below our point-by-point response.

Reviewer: 3
Dr. Zoe Kelson, University of Exeter
Comments to the Author:
This population-based surveillance study aimed to assess the effects of risk-based travel

restrictions on (i) international travel frequency, (ii) SARS-CoV-2 case importation risk, (iii) national SARS-CoV-2 incidence, and (iv) importation of SARS-CoV-2 variants in Scotland.

Reviewer comments:

"Data on the monthly number of passenger arrivals into Scottish airports, by country and airport of origin, were provided by the Civil Aviation Authority (CAA)"
and
"From June 2020, measures were introduced requiring all UK arrivals to complete a Passenger Locator Form (PLF) to support compliance with COVID-19 travel measures (23). The dataset contained weekly data for the period 28th June 2020 to 19th March 2022 on the number of PLFs submitted to Border Control and the originating countries from which passengers had travelled into Scotland"
The authors have made use of valuable data in an attempt to understand travel patterns.

We thank the reviewer for their positive comment.

"To enable like-for-like country-level comparison with PCR data, data on the Canary Islands and Madeira were merged with that of Spain and Portugal respectively in both the CAA and the PLFs. "
Can the authors please confirm that RAG status was consistently the same for these countries?

The PCR data, which was used to assess the risks and population impact of SARS-CoV-2 importations through international travel during the traffic light period, did not differentiate the Canary Islands from Spain and Maderia from Portugal. The RAG status was consistently the same for Spain and Canary Islands; they were on the Amber list for the entire duration of the period for which the RAG status was applied (17th May 2021 to 4th October 2021). While Portugal's status deviated from Madeira's from the 30th June 2021 to 4th October 2021, it is important to note that during this period, passenger numbers (CAA data) from Madeira accounted for less than 20% of the total numbers of passengers from Portugal as a whole (Portugal and Madeira combined). This has been made clearer (lines 147-151).

"Data on SARS-CoV-2 infections in Scotland were accessed from the NHS Scotland Corporate Data Warehouse (CDW). The dataset captured demographics (age, sex, postcode location) and test information (date of sample and test result). Reasons for testing included diagnostic confirmation for those with symptoms, asymptomatic testing of close contacts to support self-isolation, workplace testing as part of the NHS Test & Protect system, and post arrival testing of travellers returning from international destinations. "
Can the authors please comment on whether this self-sampled cohort can be considered to be representative of cases across Scotland?

This cohort does not comprise a sampled cohort, rather it captured all known laboratory-confirmed Scottish cases. However, we do acknowledge that despite the testing guidance there may have been non-compliance with testing guidance as well as asymptomatic or mild infections who were not tested. It was beyond the scope of this study to assess the impact of unobserved cases. This limitation was discussed, please see lines 500-510.

Can the authors please additionally consider using sample weights and modelling, based on age, sex, postcode, and various risks of exposure, to estimate scenarios of unknown cases in the (not-sampled) population across the country?

We thank the reviewer for their suggestion however given the already extensive descriptive analysis undertaken, which was the main goal of this study, it is beyond the scope to estimate unknown cases in the Scottish population. Please note this additional extensive analysis of missing data would likely substantially increase the length and coherence of our

already comprehensive paper. We believe the descriptive analysis we present is important to present and make accessible to others. We discussed the limitations (lines 500-510).

"When booking a post arrival COVID-19 test, individuals who had travelled internationally within the preceding 14 days were required to self-report the main country they had travelled to"
Can the authors please further discuss the potential for self-reporting bias?

We acknowledge that PCR tests may not capture all travellers, with compliance not quantifiable and an unknown number of individuals exempt (lines 500-502). It is therefore not possible to determine the nature of any reporting bias. We have acknowledged this limitation – see lines 502-503. Please also note that the monthly PCR test frequencies for travellers are strongly correlated with numbers of passengers into Scotland based on PLFs (line 183-185, supplementary figure S1).

"Monthly PCR test frequencies for travellers were strongly correlated with numbers of passengers into Scotland based on PLFs (Figure S1, Supplementary Material)."
and
"Although post-arrival tests were a mandatory travel policy, some exceptions were in place and compliance was not quantified (23). PLF data were therefore used to assess how representative the PCR test data was of travel frequency by destination during the traffic light period."
The authors do well to explore this.

We thank the reviewer for their positive comment.

"During the study period there were 317,570 samples from COVID-19 cases resident in Scotland that underwent SARS-CoV-2 whole genome sequencing. Samples which had a low-quality genome and samples that could not be linked to a positive SARS CoV-2 PCR test, and therefore could not be confirmed as a Scottish sample, were excluded (n=78)."
Can this cohort be considered to be representative (i.e. are case samples that underwent WGS similar to samples that did not)?

During the study period some sequence prioritisation was undertaken towards cases with a travel history and those with more severe disease. However, the distribution of age, sex, NHS board and SIMD among cases that underwent WGS was similar to total cases during the study period. This has now been added to the methods (lines 200-203) and Figure S2 (supplementary material).

"Groups at risk of importing SARS-CoV-2 through international travel were also assessed by comparing travel frequencies across demographic (age, sex, relative deprivation of residential location, as measured by the Scottish Index of Multiple Deprivation; SIMD) and geographical (territorial NHS Board) factors. "
Insightful subgroup analyses have been explored.

We thank the reviewer for their positive comment.

"An ANOVA test was used to assess differences in mean travel frequencies across demographic and geographic groups, with a p-value <0.05 applied to indicate statistical significance."
Can the authors please confirm how normality was verified for the application of parametric statistical techniques?

We thank the reviewer for the comment. Having assessed the Q-Q plot of the ANOVA model residuals, we have opted to now present the results for the more conservative Kruskal-Wallis

test due to some apparent skewness in the data for each of the factors. We have updated the methods accordingly (lines 231-236) and table 1 (pages 29-30). Please note, however, that applying the non-parametric Kruskal-Wallis test has not made any meaningful impact to the p-values, with the interpretation and conclusions from these analysis remaining unaltered.

"The potential impact of travel on the epidemiological situation in Scotland was assessed. To do so, numbers of travel-related SARS-CoV-2 cases were quantified as a proportion of all observed community cases of SARS-CoV-2 in Scotland"
Can uncertainty please be quantified and included in this assessment?

We are not sure whether the reviewer is referring to uncertainty with respect to unobserved cases, and whether the question is around travel-related cases or community cases (or both). As explained, all reported tests and cases were included but it was beyond the scope of this study to account for unobserved cases. Please also note that travel impact was assessed in a relative manner, rather than the absolute values being of primary interest, with comparisons being made across RAG groupings and travel destinations (Figure 4).

"The association between travel-based versus community-based surveillance and SARS-CoV-2 case detections was assessed in a test-negative case-control design for the traffic light system period (17th May 2021 to 30th September 2021)."
and
"Unadjusted odds ratios were initially quantified in univariate binary logistic regression models examining associations between SARS-CoV-2 infection and travel status, age group (0-19y, 20-39y, 40-59y, 60-79y, 80y+), sex (male versus female), month (May, June, July, August, September) and NHS Board (fourteen territorial health boards). Multivariable mixed-effects logistic regression modelling was then used to quantify the relative odds of SARS-CoV-2 case detection adjusting for age group, sex, and calendar month as fixed effects and NHS Board location as a random effect. Statistical interactions were assessed for all factors that were significant in the final multivariable model, with a p-value less than 0.05 indicating statistical significance."
and
"The glm function was applied for single effect statistical models and the lme4 (1.1-27.1 package) for mixed-effect models"
Appropriate modelling methods have been applied.

We thank the reviewer for their positive comment.

"Table 2. Investigating risk factors for SARS-CoV-2 infection during the period of the traffic light system (17th May 2021 to 30th September 2021) in Scotland."
Can the authors please clarify the outcome variable in these models (i.e. OR for what)?

The outcome variable is SARS-CoV-2 case detection. This has been made clearer in the title for Table 2 (line 736).

How to interpret this table is currently unclear, can the authors please provide further clarification?

We are not sure which aspect of the table the reviewer has found unclear. In the results section we have now added text to make it clear that the adjusted odd ratio controls for age group, sex, month and geographic location (line 372-374). We have also added further detail to the Table 2 notes to make it clear the unadjusted odd ratio is estimated from single factor models and that the adjusted odd ratios are estimated from a model that adjusts for all the factors in Table 2 (lines 740-744). We hope this addressed the comment.

"Our study has a number of limitations. Firstly, laboratory surveillance data may not capture all travellers; an unknown number of individuals were exempt from testing and compliance is not quantifiable. While Scotland had the autonomy to implement its own travel policies during the study period these aligned with the rest of the UK. There was unrestricted travel within the UK and individuals arriving elsewhere in the UK with onward travel to Scotland were not captured in traveller tests. Secondly, deductions of SARS-CoV-2 infection risks across travel-based and community-based surveillance groups must be made with caution. The greater odds of detecting SARS CoV-2 cases through community surveillance may be explained by the targeted testing of symptomatic individuals and known close contacts of confirmed cases.
Furthermore, those entering the UK were required to take a pre-departure test during the traffic light period, so the proportion of cases in this group is expected to reflect the risk of SARS-CoV-2 importation - combining the risks of a travel-associated infection and testing negative prior to departure. This should not preclude the validity of comparing SARS-CoV-2 case frequency over time, by travel destination, and across demographic and geographic groups. Thirdly, case misclassifications may have arisen, with some over-ascription of infections to the period of international travel (the acquisition of infection before or after travel cannot be ruled out, including from household transmission). Fourthly, in the absence of a suitable control population or period, our study did not assess the reduction in SARS-CoV-2 case incidence in Scotland attributable to the traffic light system."
A discussion on the study limitations has been provided by the authors.

We thank the reviewer for their positive comment.

Can the authors please do more to model uncertainties in an attempt to account for potential biases in the data and estimate the number of unknown cases under different scenarios and assumptions?

As explained above the community cases include all known Scottish cases and we have highlighted the different reasons for testing in the community and among travellers (lines 160-163) and the limitations (506-510). We have carried out an extensive descriptive analysis but developing methods to estimate or model unknown cases was beyond the scope of this study.

"Our findings suggest that country-specific post-arrival screening undertaken in Scotland did not prohibit the public health impact of COVID-19 in Scotland."
and
"Our findings show that risk-based post-arrival screening undertaken in Scotland did not, in practice, prohibit the importation of SARS-CoV-2 cases, or the establishment of SARS-CoV-2 VOC in the Scottish community, arising through international travel."
Of note, the aim of travel restrictions, the RAG system, and post-arrival screening, might not have been to prohibit international risks, but instead to reduce these risks to a level where health systems were better able to manage the speed of spread and volume of cases.
For a more nuanced evaluation, can the authors please consider comparing the outcomes to other hypothetical scenarios (e.g. what might be expected if travel was permitted without the RAG system and post-arrival screening in place, or a different suggestion for travel restriction and monitoring was introduced, or alternatively no travel was permitted at all)?

We have discussed the lack of comparative time periods or populations to assess the impact of the RAG system on reducing case incidence in the Scottish community as a part of this study (lines 519-521). However, we compared the odds of SARS-CoV-2 detection across the different RAG levels (which had different restrictions in place) and show higher odds of SARS-CoV-2 detection among travellers required to adhere to greater restrictions (red compared to amber and amber compared to green). In addition, modelling the population

dynamics is an involved and different research question and therefore beyond the scope of this study.

"Travel rates likely contributed to patterns of high SARS-CoV-2 case importation and population impact. "
Can the authors please re-consider whether this conclusion can be inferred from the scope of this study?
How are 'high' and 'impact' defined here?

We have edited this conclusion to now read ‘Travel rates likely contributed to patterns of SARS-CoV-2 case importation and population incidence.’ (lines 46-47).

Thanks for providing a copy of the STROBE checklist.

We thank the reviewer for their positive comment.

In addition to the above, we have made some minor edits throughout for readability.
